# A Framework for Modeling and Interpreting Patient Subgroups Applied to Hospital Readmission: Visual Analytical Approach

**DOI:** 10.2196/37239

**Published:** 2022-12-07

**Authors:** Suresh K Bhavnani, Weibin Zhang, Shyam Visweswaran, Mukaila Raji, Yong-Fang Kuo

**Affiliations:** 1 School of Public and Population Health University of Texas Medical Branch Galveston, TX United States; 2 Department of Biomedical Informatics University of Pittsburgh Pittsburgh, PA United States; 3 Division of Geriatric Medicine Department of Internal Medicine University of Texas Medical Branch Galveston, TX United States

**Keywords:** visual analytics, Bipartite Network analysis, hospital readmission, precision medicine, modeling, Medicare

## Abstract

**Background:**

A primary goal of precision medicine is to identify patient subgroups and infer their underlying disease processes with the aim of designing targeted interventions. Although several studies have identified patient subgroups, there is a considerable gap between the identification of patient subgroups and their modeling and interpretation for clinical applications.

**Objective:**

This study aimed to develop and evaluate a novel analytical framework for modeling and interpreting patient subgroups (MIPS) using a 3-step modeling approach: *visual analytical* modeling to automatically identify patient subgroups and their co-occurring comorbidities and determine their statistical significance and clinical interpretability; *classification* modeling to classify patients into subgroups and measure its accuracy; and *prediction* modeling to predict a patient’s risk of an adverse outcome and compare its accuracy with and without patient subgroup information.

**Methods:**

The MIPS framework was developed using bipartite networks to identify patient subgroups based on frequently co-occurring high-risk comorbidities, multinomial logistic regression to classify patients into subgroups, and hierarchical logistic regression to predict the risk of an adverse outcome using subgroup membership compared with standard logistic regression without subgroup membership. The MIPS framework was evaluated for 3 hospital readmission conditions: chronic obstructive pulmonary disease (COPD), congestive heart failure (CHF), and total hip arthroplasty/total knee arthroplasty (THA/TKA) (COPD: n=29,016; CHF: n=51,550; THA/TKA: n=16,498). For each condition, we extracted cases defined as patients readmitted within 30 days of hospital discharge. Controls were defined as patients not readmitted within 90 days of discharge, matched by age, sex, race, and Medicaid eligibility.

**Results:**

In each condition, the visual analytical model identified patient subgroups that were statistically significant (*Q*=0.17, 0.17, 0.31; *P*<.001, <.001, <.05), significantly replicated (Rand Index=0.92, 0.94, 0.89; *P*<.001, <.001, <.01), and clinically meaningful to clinicians. In each condition, the classification model had high accuracy in classifying patients into subgroups (mean accuracy=99.6%, 99.34%, 99.86%). In 2 conditions (COPD and THA/TKA), the hierarchical prediction model had a small but statistically significant improvement in discriminating between readmitted and not readmitted patients as measured by net reclassification improvement (0.059, 0.11) but not as measured by the C-statistic or integrated discrimination improvement.

**Conclusions:**

Although the visual analytical models identified statistically and clinically significant patient subgroups, the results pinpoint the need to analyze subgroups at different levels of granularity for improving the interpretability of intra- and intercluster associations. The high accuracy of the classification models reflects the strong separation of patient subgroups, despite the size and density of the data sets. Finally, the small improvement in predictive accuracy suggests that comorbidities alone were not strong predictors of hospital readmission, and the need for more sophisticated subgroup modeling methods. Such advances could improve the interpretability and predictive accuracy of patient subgroup models for reducing the risk of hospital readmission, and beyond.

## Introduction

### Overview

A wide range of studies [1-9] on topics ranging from molecular to environmental determinants of health have shown that most humans tend to share a subset of characteristics (eg, comorbidities, symptoms, or genetic variants), forming distinct patient subgroups. A primary goal of precision medicine is to identify such patient subgroups, and to infer their underlying disease processes to design interventions targeted at those processes [2,10]. For example, recent studies on complex diseases such as breast cancer [3,4], asthma [5-7], and COVID-19 [11] have revealed patient subgroups, each with different underlying mechanisms precipitating the disease and therefore each requiring different interventions.

However, there is a considerable gap between the identification of patient subgroups and their modeling and interpretation for clinical applications. To bridge this gap, we developed and evaluated a novel analytical framework called modeling and interpreting patient subgroups (MIPS) using a 3-step modeling approach: (1) identification of patient subgroups, their frequently co-occurring characteristics, and their risk of adverse outcomes; (2) classification of a new patient into one or more subgroups; and (3) prediction of an adverse outcome for a new patient informed by subgroup membership. We evaluated MIPS on 3 data sets related to hospital readmission, which helped pinpoint the strengths and limitations of MIPS. Furthermore, the results provided implications for improving the interpretability of patient subgroups in large and dense data sets, and for the design of clinical decision support systems to prevent adverse outcomes such as hospital readmissions.

### Identification of Patient Subgroups

Patients have been divided into subgroups using (1) investigator-selected variables such as race for developing hierarchical regression models [12] or assigning patients to different arms of a clinical trial, (2) existing classification systems such as the Medicare Severity-Diagnosis Related Group [13] to assign patients to a disease category for purposes of billing, and (3) computational methods such as classification [14-16] and clustering [5,17] to discover patient subgroups from data (also referred to as *subtypes* or *phenotypes* depending on the condition and variables analyzed).

Several studies have used a wide range of computational methods to identify patient subgroups, each with critical trade-offs. Some studies have used *combinatorial* approaches [18] (identifying all pairs, all triples, etc), which although intuitive, can lead to a combinatorial explosion (eg, enumerating combinations of the 31 Elixhauser comorbidities would lead to 2^31^ or 2147483648 combinations), with most combinations that do not incorporate the full range of symptoms (eg, the most frequent pair of symptoms ignores which other symptoms exist in the profile of patients with that pair). Other studies have used *unipartite* clustering methods [16,17] (clustering patients or comorbidities but not both together), such as k-means and hierarchical clustering. Furthermore, dimensionality-reduction methods such as principal component analysis used with unipartite clustering methods have been used to identify clusters of frequently co-occurring comorbidities [18-24]. However, such methods have well-known limitations, including the requirement of inputting user-selected parameters (eg, similarity measures and the number of expected clusters) and the lack of a quantitative measure to describe the quality of the clustering (critical for measuring the statistical significance of the clustering). Furthermore, because these methods are unipartite, there is no agreed-upon method for identifying the patient subgroup defined by a cluster of variables, and vice versa.

More recently, bipartite network analysis [25] has been used to address these limitations by automatically identifying *biclusters,* consisting of patients and characteristics simultaneously. This method takes as input any data set, such as patients and their comorbidities, and outputs a quantitative and visual description of biclusters (containing both patient subgroups and their frequently co-occurring comorbidities). The quantitative output generates the number, size, and statistical significance of the biclusters [26-28], and the visual output displays the quantitative information of the biclusters through a network visualization [29-31]. Bipartite network analysis therefore enables (1) the automatic identification of biclusters and their significance and (2) the visualization of the biclusters critical for their clinical interpretability. Furthermore, the attributes of patients in a subgroup can be used to measure the subgroup risk for an adverse outcome, develop classification models for classifying a new patient into one or more of the subgroups, and develop prediction models that use subgroup membership for measuring the risk of an adverse outcome for the classified patient.

However, although several studies [11,28,32-38] have demonstrated the usefulness of bipartite networks for the identification and clinical interpretation of patient subgroups, there has been no systematic attempt to integrate them with classification and prediction modeling, which is a critical step toward their clinical application. Therefore, we leveraged the advantages of a bipartite network to develop the MIPS framework with the goal of bridging the gap between the identification of patient subgroups, and their modeling and interpretation for future clinical applications.

### The Need for Modeling and Interpreting Patient Subgroups in Hospital Readmission

An estimated 1 in 5 elderly patients (more than 2.3 million Americans) is readmitted to a hospital within 30 days of discharge [39]. Although many readmissions are unavoidable, an estimated 75% of readmissions are unplanned and mostly preventable [40], imposing a significant burden in terms of mortality, morbidity, and resource consumption. Across all conditions, unplanned readmissions in the United States cost approximately US $17 billion [40], making them an ineffective use of costly resources. Consequently, hospital readmission is closely scrutinized as a marker for poor quality of care by organizations such as the Centers for Medicare & Medicaid Services (CMS) [41].

To address this epidemic of hospital readmission, CMS sponsored the development of models to predict the patient-specific risk of readmission in specific index conditions such as chronic obstructive pulmonary disease (COPD) [42], congestive heart failure (CHF) [43], and total hip arthroplasty/total knee arthroplasty (THA/TKA) [44]. As numerous studies have shown that almost two-thirds of older adults have 2 or more comorbid conditions with a heightened risk of adverse health outcomes [18], the independent variables in the CMS models included prior comorbidities (as recorded in Medicare claims data) and demographics (age, sex, and race). However, although prior studies have shown the existence of subgroups among patients with hospital readmission [28], none of the CMS models have incorporated patient subgroups. The identification and inclusion of patient subgroups could improve the accuracy of predicting hospital readmission for a patient, in addition to enabling the design of interventions targeted at each patient subgroup to reduce the risk of readmission. Therefore, we used the MIPS framework to model and interpret patient subgroups in hospital readmission and tested its generality across the 3 index conditions. Furthermore, to enable a head-to-head comparison with existing CMS predictive models, we used the same independent variables as were used in those models, in addition to patient subgroup membership when developing our prediction models.

## Methods

### Overview

**Figure 1:**
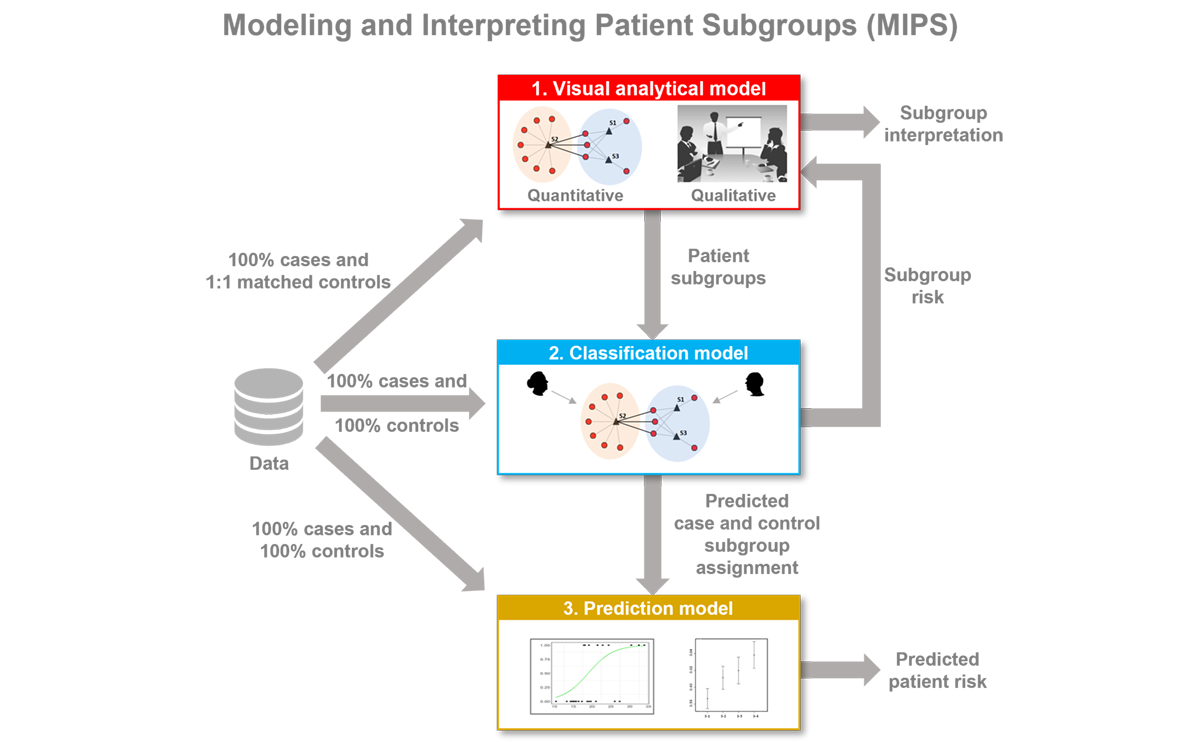
Inputs and outputs for the 3-step modeling in MIPS consisting of the visual analytical model, classification model, and prediction model. MIPS: Modeling and Interpreting Patient Subgroups.

Figure 1 provides a conceptual description of the data inputs and outputs from the 3-step modeling in MIPS. The visual analytical model identifies patient subgroups and visualizes them through a network. The classification model determines the subgroup membership for cases and controls. These subgroup memberships are then used to measure the risk for readmission within each subgroup based on the proportion of cases and juxtaposed with the respective subgroup visualization to enable clinicians to interpret the readmitted patient subgroups. Finally, the prediction model uses the subgroup membership assignment of cases and controls to determine the readmission risk of a patient. Multimedia Appendix 1 [16,23,25-31,45,46] provides a summary of the inputs, methods, and outputs for each model.

### Data Description

#### Study Population

We analyzed patients hospitalized for COPD, CHF, or THA/TKA. We selected these 3 index conditions because (1) hospitalizations for each of these conditions are highly prevalent in older adults [39], (2) hospitals report very high variations in their readmission rates [39], and (3) there exist well-tested readmission prediction models for each of these conditions that do not consider patient subgroups [42-44,47,48].

Data for these 3 index conditions were extracted from the Medicare insurance claims data set. In 2019, Medicare provided health insurance to approximately 64.4 million Americans, of whom 55.5 million were older Americans (≥65 years) [49]. Furthermore, 94% of noninstitutionalized older Americans were covered by Medicare [50], with eligible claims received from 6204 medical institutions across the United States, and is therefore one of the few data sets that is highly representative of older Americans and their care.

For each index condition, we used the same inclusion and exclusion criteria that were used to develop the CMS models but with the most recent years (2013-2014) provided by Medicare when we started the project. We extracted all patients who were admitted to an acute care hospital between July 2013 and August 2014 with a principal diagnosis of the index condition, were aged ≥66 years, and were enrolled in both Medicare parts A and B fee-for-service plans 6 months before admission. Furthermore, we excluded patients who were transferred from other facilities, died during hospitalization, or transferred to another acute care hospital. Similar to the CMS models, we selected the first admission for patients with multiple admissions during the study period, and we did not use data from Medicare Part D (related to prescription medications).

Multimedia Appendix 2 [40,44] describes (1) the International Classification of Diseases, Ninth Version, codes for each of the 3 index conditions selected for analysis and (2) the inclusion and exclusion criteria used to extract cases and controls for COPD, CHF, and THA/TKA; the respective numbers of patients extracted at each step; and how we addressed the small incidence of missing data. Each modeling method used relevant subsets of these data, as described in the Analytical and Evaluation Approach section.

#### Variables

The independent variables consisted of comorbidities and patient demographics (age, sex, and race). Comorbidities common in older adults were derived from 3 established comorbidity indices: Charlson Comorbidity Index [51], Elixhauser Comorbidity Index [52], and the Center for Medicare and Medicare Services Condition Categories used in the CMS readmission models [53] (the variables in the CMS models varied across the index conditions). As these indices had overlapping comorbidities, we derived a union of them, which was verified by the clinician stakeholders. They recommended that we also include the following additional variables, as they were pertinent to each index condition: COPD (history of sleep apnea and mechanical ventilation), CHF (history of coronary artery bypass graft surgery), and THA/TKA (congenital deformity of the hip joint and posttraumatic osteoarthritis). For each patient in our cohort, we extracted these comorbidities and variables from the physicians, outpatient, and inpatient Medicare claims data in the 6 months before (to guard against miscoding) and on the day of the index admission. The dependent variable (outcome) was whether a patient with an index admission (COPD, CHF, or THA/TKA) had an unplanned readmission to an acute care hospital within 30 days of discharge as was recorded in the Medicare Provider Analysis and Review file (inpatient claims) in the Medicare database.

### Analytical and Evaluation Approach

#### Visual Analytical Modeling

The goal of visual analytical modeling was to identify and interpret biclusters of readmitted patients (cases), consisting of patient subgroups and their most frequently co-occurring comorbidities. The data used to build the visual analytical model in each index condition consisted of randomly dividing 100% of the cases into training (50%) and replication (50%) data sets (we use the term *replication* to avoid confusion with the term *validation* typically used in classification and prediction models). For feature selection, we extracted an equal number of 1:1 matched controls based on age, sex, race, and ethnicity, and Medicaid eligibility [45]. These data were analyzed for each index condition using the following steps (Multimedia Appendix 1 provides additional details for each step):

1. *Model training*: to train the visual analytical model, we used feature selection to identify the set of comorbidities that were univariably significant in both the training and replication data sets and used bicluster modularity maximization [26,27] to identify the number, members, and significance of biclusters in the training data set.
2. *Model replication*: to test the replicability of the biclusters, we repeated the bicluster analysis on the replication data set and used the Rand Index (RI) [46] to measure the degree and significance of similarity in comorbidity co-occurrence between the 2 data sets.
3. *Model interpretation*: to enable clinical interpretation of the patient subgroups, we used the *Fruchterman-Reingold* [29] and *ExplodeLayout* [30,31] algorithms to visualize the network. Furthermore, based on a request from our clinician stakeholder team, for each bicluster, we ranked and displayed the comorbidity labels with their univariable odds ratios (ORs) for readmission (obtained from the feature selection mentioned earlier) and juxtaposed the readmission risk of the bicluster (obtained from the classification step discussed in the next section) onto the network visualization. Clinician stakeholders were asked to use the visualization to interpret patient subgroups, their mechanisms, and potential interventions to reduce the risk of readmission.

#### Classification Modeling

The goal of classification modeling was to classify all cases and controls from the entire Medicare data set into the biclusters identified from the visual analytical model. The resulting bicluster membership for all cases and controls was designed to (1) develop the predictive modeling described in the next section and (2) measure the risk of each subgroup to enable clinical interpretation of the patient subgroups. The training data set in each condition consisted of a random sample of 75% cases with their subgroup membership (output of the visual analytical modeling) and an internal validation data set consisting of randomly selected 25% of the cases (with subgroup membership used to validate the model). These data were used to develop and use classification models for each index condition using the following steps (Multimedia Appendix 1 provides additional details for each step):

1. *Model training*: to train the classifier, we used multinomial logistic regression [16] with independent variables consisting of comorbidities (identified through feature selection). The accuracy of the trained model was measured by calculating the percentage of times the model correctly classified the cases into subgroups using the highest predicted probability across the subgroups.
2. *Model internal validation*: to internally validate the classifier, we randomly split these data into training (75%) and testing (25%) data sets 1000 times. For each iteration, we trained a model using the training data set and measured its accuracy using the testing data set. This was done by predicting subgroup membership using the highest predicted probability among all the subgroups. The overall predicted accuracy was estimated by calculating the mean accuracy across the 1000 models.
3. *Model application*: to generate data for the visual analytical and prediction models, the classifier was used to classify 100% of cases and controls from our entire Medicare data set (July 2013-August 2014). The resulting classified data were used to measure the risk of each subgroup (juxtaposed onto the network visualization to enable clinical interpretation) and to conduct the following prediction modeling.

#### Prediction Modeling

The goal of prediction modeling was to predict the risk of readmission for a patient, taking into consideration subgroup membership. The data used to build the prediction models consisted 100% of cases and 100% of controls, with subgroup membership generated from the classification modeling. These data were randomly spilt into training (75%) and internal validation (25%) data sets. These data were used to train, internally validate, and compare the prediction models in each index condition using the following steps (Multimedia Appendix 1 provides additional details for each step):

1. *Model training*: to train the prediction model, we used binary logistic regression for developing a Standard Model (without subgroup membership, similar to the CMS models) and a Hierarchical Model (with subgroup membership). The independent variables for both models consisted of comorbidities (identified through feature selection) and demographics, and the outcome was 30-day unplanned readmission (yes vs no).
2. *Model internal validation*: to internally validate the models, we used the internal validation data set to measure discrimination (C-statistic) and calibration (calibration-in-the-large and calibration slope).
3. *Model comparisons*: to compare the accuracy of the Standard and Hierarchical Models, we used the chi-squared test to compare their C-statistics. Furthermore, to examine how the Standard Model was applied to each subgroup, we measured the C-statistics of the Standard Model applied to each subgroup separately. Finally, because both these models used comorbidities selected through feature selection, they differed from the set of comorbidities used in the published CMS models. Therefore, to perform a head-to-head comparison with the published CMS models (COPD [42], CHF [43], and THA/TKA [44]), we developed a logistic regression model using the independent variables from the published CMS model (CMS Standard Model) and compared it to the same model, but which also included subgroup membership (CMS Hierarchical Model). Similar to these comparisons, we used the chi-squared test to compare the C-statistics of the CMS standard and the CMS Hierarchical Models and additionally measured the differences between the models using net reclassification improvement (NRI) and integrated discrimination improvement (IDI).

### Ethics Approval

Medicare data were analyzed using a CMS data-use agreement (CMS DUA RSCH-2017-51404) and approved by the University of Texas Medical Branch Institutional Review Board (16-0361).

## Results

### Data

Table 1 summarizes the number of cases and controls used to develop the 3 models for each condition.

**Table 1:** Training and replication/validation data sets used to develop the three models in each of the 3 index conditions.

Model		Training	Replication/validation	Total
**Visual analytical^a^ (cases/controls)**				
	Chronic obstructive pulmonary disease (COPD)	14,508/14,508	14,508/14,508	29,016/29,016
	Congestive heart failure (CHF)	25,775/25,775	25,775/25,775	51,550/51,550
	Total hip arthroplasty/total knee arthroplasty (THA/TKA)	8249/8249	8249/8249	16,498/16,948
**Classification (cases)**				
	COPD	10,842	3615	14,457
	CHF	19,254	6418	25,672
	THA/TKA	5257	1753	7010
**Prediction (cases/controls)**				
	COPD	21,692/117,839	7334/39,176	29,026/157,015
	CHF	38,728/183,093	12,845/61,095	51,573/244,188
	THA/TKA	12,376/255,203	41,44/85,049	16,520/340,252

^a^The visual analytical models used 1:1 matched controls for the feature selection, and used only cases for the bipartite networks to analyze heterogeneity in readmission. The numbers shown for the visual analytical models are before removing patients with no comorbidities. The resulting cases-only data sets were used for the classification modelling as shown.

### Visual Analytical Modeling

#### Overview

Visual analytical modeling of readmitted patients in all 3 index conditions produced statistically and clinically significant patient subgroups and their most frequently co-occurring comorbidities, which were significantly replicated. We report the results for each index condition.

#### COPD Visual Analytical Model

The inclusion and exclusion selection criteria (Multimedia Appendix 2) resulted in a training data set (n=14,508 matched case-control pairs, of which 51 patient pairs had no dropped comorbidities) and a replication data set (n=14,508 matched case-control pairs, of which 51 patient pairs had no dropped comorbidities), matched by age, sex, race, and Medicaid eligibility (a proxy for economic status). The feature selection method (Multimedia Appendix 3) used 45 unique comorbidities identified from a union of the 3 comorbidity indices, plus 2 condition-specific comorbidities. Of these, 3 were removed because of <1% prevalence. Of the remaining comorbidities, 30 survived significance and replication testing using Bonferroni correction. The visual analytical model used these surviving comorbidities (d=30), and readmitted patients with COPD with at least one of these comorbidities (n=14,457).

**Figure 2:**
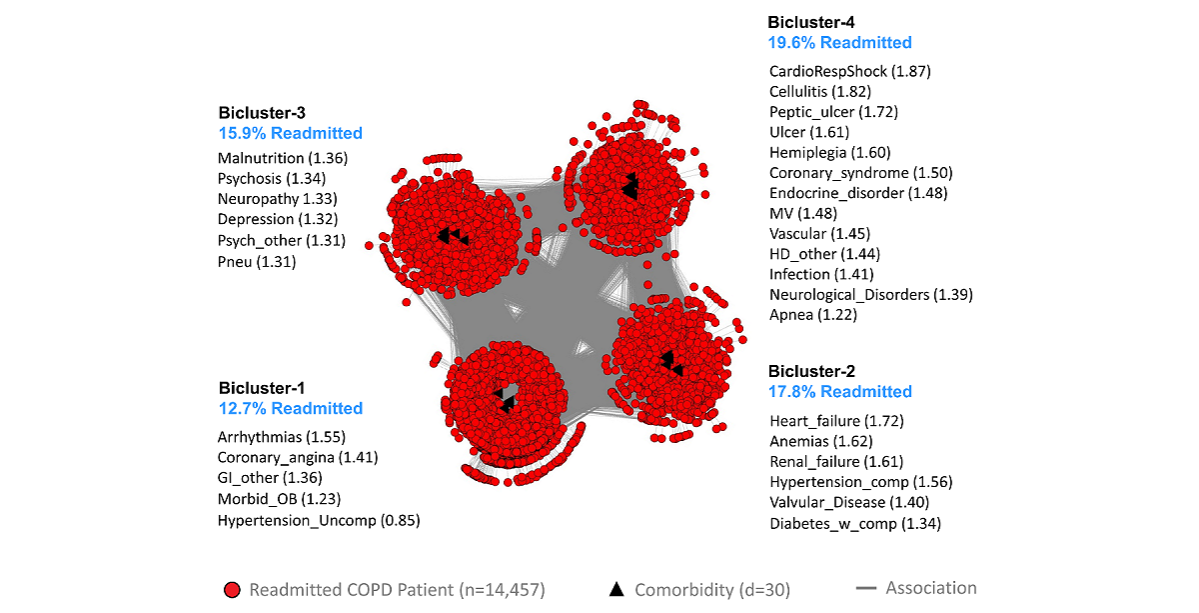
The chronic obstructive pulmonary disease (COPD) visual analytical model showing 4 biclusters consisting of patient subgroups and their most frequently co-occurring comorbidities (whose labels are ranked by their univariable odds ratios, shown within parentheses) and their risk of readmission (shown in blue text). GI: Gastrointestinal disorders; HD: Heart disease; MV: History of mechanical ventilation.

As shown in Figure 2, bipartite network analysis identified 4 biclusters, each representing a subgroup of readmitted patients with COPD and their most frequently co-occurring comorbidities. Biclustering had significant modularity (*Q*=0.17; *z*=7.3; *P*<.001) and significant replication (RI=0.92; *z*=11.62; *P*<.001) of comorbidity co-occurrence. Furthermore, as requested by the clinician stakeholders, we juxtaposed a ranked list of comorbidities based on their ORs for readmission in each bicluster, in addition to the risk for each patient subgroup.

The pulmonologist inspected the visualization and noted that the readmission risk of the patient subgroups had a wide range (12.7%-19.6%) with clinical (face) validity. Furthermore, the co-occurrence of comorbidities in each patient subgroup was clinically meaningful with interpretations for each subgroup. Subgroup-1 had a low disease burden, with uncomplicated hypertension leading to the lowest risk (12.7%). This subgroup represented patients with early organ dysfunction and would benefit from using checklists such as regular monitoring of blood pressure in predischarge protocols to reduce the risk of readmission. Subgroup-3 had mainly psychosocial comorbidities, which could lead to aspiration precipitating pneumonia, leading to an increased risk for readmission (15.9%). This subgroup would benefit from early consultation with specialists (eg, psychiatrists, therapists, neurologists, and geriatricians) who have expertise in psychosocial comorbidities, with a focus on the early identification of aspiration risks and precautions. Subgroup-2 had diabetes with complications, renal failure, and heart failure and therefore had higher disease burden, leading to an increased risk of readmission (17.8%) compared with Subgroup-1. This subgroup had metabolic abnormalities with greater end-organ dysfunction and would therefore benefit from case management by advanced practice providers (eg, nurse practitioners) with rigorous adherence to established guidelines to reduce the risk of readmission. Subgroup-4 had diseases with end-organ damage, including gastrointestinal disorders, and therefore had the highest disease burden and risk for readmission (19.6%). This subgroup would also benefit from case management with rigorous adherence to established guidelines to reduce the risk of readmission. Furthermore, as patients in this subgroup typically experience complications that could impair their ability to make medical decisions, they should be provided with early consultation with a palliative care team to ensure that care interventions align with patients’ preferences and values.

#### CHF Visual Analytical Model

**Figure 3:**
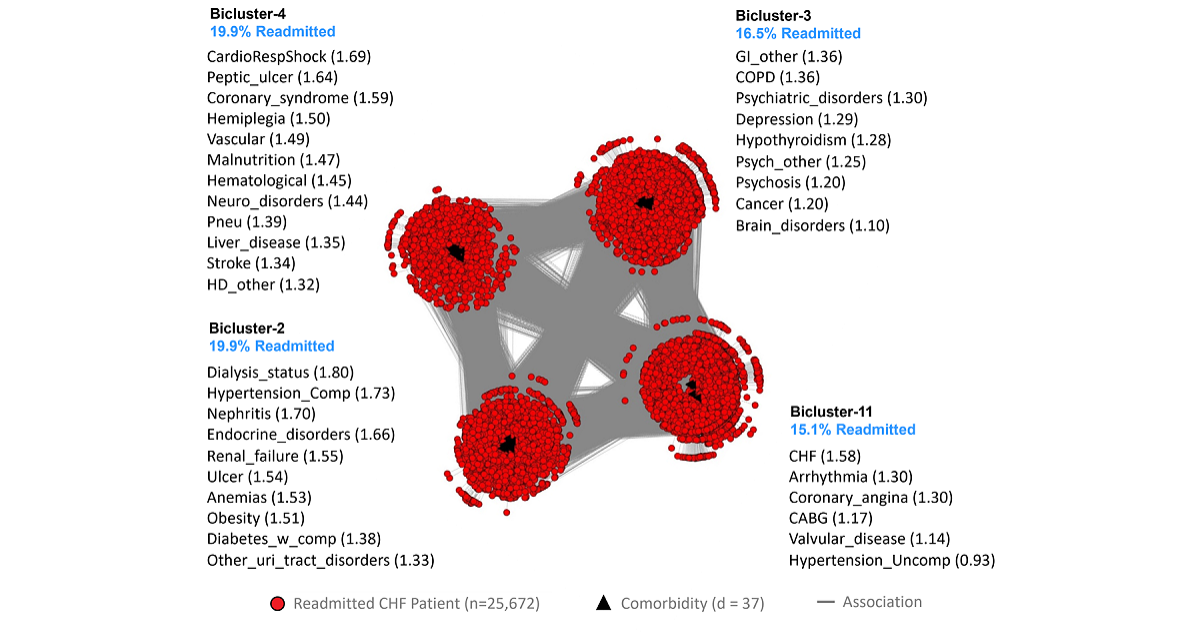
The congestive heart failure (CHF) visual analytical model showing 4 biclusters consisting of patient subgroups and their most frequently co-occurring comorbidities (whose labels are ranked by their univariable odds ratios, shown within parentheses) and their risk of readmission (shown in blue text). CABG: History of coronary artery bypass graft surgery; COPD: Chronic obstructive pulmonary disease; GI: Gastrointestinal disorders; HD: Heart disease.

The inclusion and exclusion selection criteria (Multimedia Appendix 2) resulted in a training data set (n=25,775 matched case-control pairs, of which 103 patient pairs with no dropped comorbidities) and a replication data set (n=25,775 matched case-control pairs, of which 104 patient pairs with no dropped comorbidities), matched by age, sex, race, and Medicaid eligibility (a proxy for economic status). The feature selection method (Multimedia Appendix 3) used 42 unique comorbidities identified from a union of the 3 comorbidity indices plus 1 condition-specific comorbidity. Of these, 1 comorbidity was removed because of <1% prevalence. Of those remaining, 37 survived the significance and replication testing with the Bonferroni correction. The visual analytical model (Figure 3) used these surviving comorbidities (d=37) and cases consisting of readmitted patients with CHF, with at least one of those comorbidities (n=25,672). As shown in Figure 3, the bipartite network analysis of the CHF cases identified 4 biclusters, each representing a subgroup of readmitted patients with CHF and their most frequently co-occurring comorbidities. The analysis revealed that the biclustering had significant modularity (*Q*=0.17; *z*=8.69; *P*<.001) and significant replication (RI=0.94; *z*=17.66; *P*<.001) of comorbidity co-occurrence. Furthermore, as requested by the clinicians, we juxtaposed a ranked list of comorbidities based on their ORs for readmission in each bicluster, in addition to the risk for each of the patient subgroups.

The geriatrician inspected the visualization and noted that the readmission risk of the patient subgroups, ranging from 15.1% to 19.9%, was wide, with clinical (face) validity. Furthermore, the co-occurrence of comorbidities in each patient subgroup was clinically significant. Subgroup-1 had chronic but stable conditions and therefore had the lowest risk for readmission (15.1%). Subgroup-3 had mainly psychosocial comorbidities but was not as clinically unstable or fragile compared with Subgroup-2 and Subgroup-4, and therefore had medium risk (16.6%). Subgroup-2 had severe chronic conditions, making them clinically fragile (with potential benefits from early palliative and hospice care referrals), and were therefore at high risk for readmission if nonpalliative approaches were used (19.9%). Subgroup-4 had severe acute conditions that were also clinically unstable, associated with substantial disability and care debility and therefore at high risk for readmission and recurrent intensive care unit use (19.9%).

#### THA/TKA Visual Analytical Model

**Figure 4:**
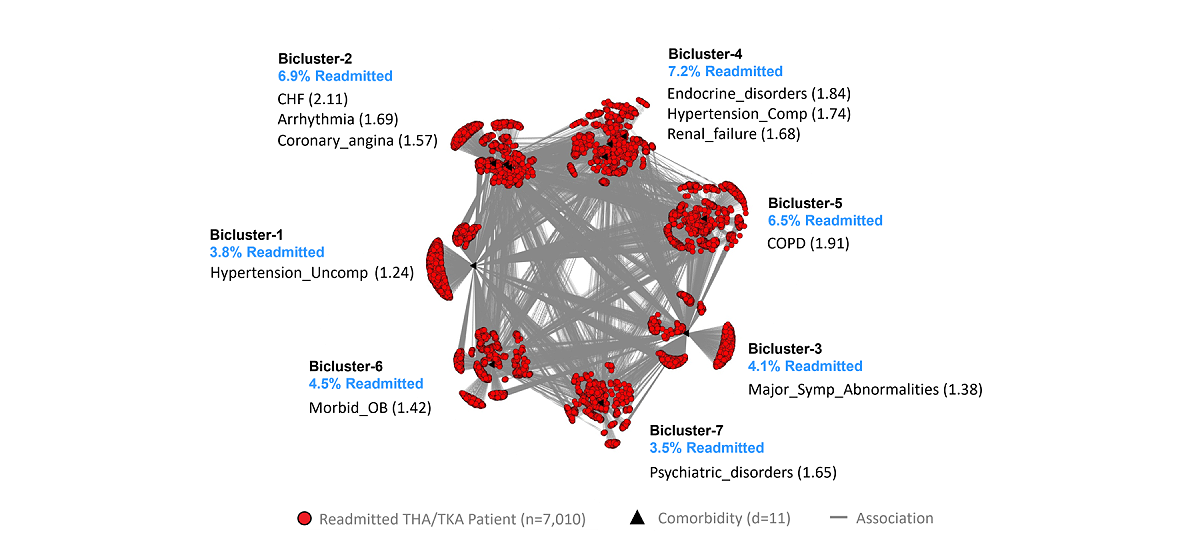
The total hip arthroplasty/total knee arthroplasty (THA/TKA) visual analytical model showing 4 biclusters consisting of patient subgroups and their most frequently co-occurring comorbidities (whose labels are ranked by their univariable odds ratios, shown within parentheses) and their risk for readmission (shown in blue text). CHF: Congestive heart failure; COPD: Chronic obstructive pulmonary disease; OB: Obesity.

The inclusion and exclusion selection criteria (Multimedia Appendix 2) resulted in a training data set (n=8249 matched case-control pairs, of which 1239 patient pairs had no dropped comorbidities) and a replication data set (n=8249 matched case-control pairs, of which 1264 patient pairs had no dropped comorbidities), matched by age, sex, race, and Medicaid eligibility (a proxy for economic status). Feature selection (Multimedia Appendix 3) used 39 unique comorbidities identified from the 3 comorbidity indices plus 2 condition-specific comorbidities. Of these, 11 comorbidities were excluded because of <1% prevalence. Of the remaining, 11 comorbidities survived significance and replication testing with the Bonferroni correction. The visual analytical model (Figure 4) used these surviving comorbidities (d=11) and cases consisting of readmitted patients with at least one of those comorbidities (n=7010).

As shown in Figure 4, the bipartite network analysis of THA/TKA cases identified 7 biclusters, each representing a subgroup of readmitted patients with THA/TKA and their most frequently co-occurring comorbidities. The analysis revealed that biclustering had significant modularity (*Q*=0.31; *z*=2.52, *P*=.01), and significant replication (RI=0.89; *z*=3.15; *P*=.002) of comorbidity co-occurrence. Furthermore, as requested by the clinician stakeholders, we juxtaposed a ranked list of comorbidities based on their ORs for readmission in each bicluster, in addition to the risk for each patient subgroup.

The geriatrician inspected the network and noted that patients with total knee arthroplasty, in general, were healthier than patients with total hip arthroplasty. Therefore, the network was difficult to interpret when the 2 index conditions were merged together. Although our analysis was constrained because we used the conditions defined by CMS, these results nonetheless suggest that the interpretations did not suffer from a *confirmation bias* (manufactured interpretations to fit the results). However, he noted that the range of readmission risk had clinical (face) validity. Furthermore, Subgroup-2, Subgroup-4, and Subgroup-5 had more severe comorbidities related to the lung, heart, and kidney and therefore had a higher risk for readmission compared with Subgroup-1, Subgroup-6, and Subgroup-7, which had less severe comorbidities and therefore had a lower risk for readmission. In addition, Subgroup-2, Subgroup-5, Subgroup-6, and Subgroup-7 would benefit from chronic care case management from advanced practice providers (eg, nurse practitioners). Finally, Subgroup-2 and Subgroup-5 would benefit from using well-established guidelines for CHF and COPD, Subgroup-7 would benefit from mental health care and management of psychosocial comorbidities, and Subgroup-6 would benefit from care for obesity and metabolic disease management.

### Classification Modeling

#### Overview

The classification model used multinomial logistic regression for each index condition (Multimedia Appendix 4 for the model coefficients) to predict the membership of patients using subgroups (identified from the aforementioned visual analytical models). The results revealed that in each index condition, the classification model had high accuracy in classifying all the cases in the full data set (training data set used in the visual analytical modeling). Similarly, the internal validation results using a 75%:25% split of this data set also had a high classification accuracy (Table 2 with classification accuracy divided into quantiles). We report the results for each index condition.

**Table 2:** Internal validation results showing the percentage of chronic obstructive pulmonary disease (COPD) congestive heart failure (CHF), and total hip arthroplasty/total knee arthroplasty (THA/TKA) patients correctly-assigned to a subgroup by the classification models in each condition.

Models		Quantiles						Summary, mean (SD; range)
		Q 0.025	Q 0.25	Q 0.50	Q 0.75	Q 0.975		
**COPD**								
	Training (n=10842)	99.90	100.00	100.00	100.00	100.00	100 (0.02; 99.7-100)	
	Testing (n=3615)	99.30	99.40	99.60	99.60	99.80	99.6 (0.15; 99.1-100)	
**CHF**								
	Training (n=19254)	99.40	99.50	99.60	99.60	99.80	99.57 (0.11; 99-99.9)	
	Testing (n=6418)	99.00	99.30	99.30	99.40	99.60	99.34 (0.15; 98.7-99.7)	
**THA/TKA**								
	Training (n=5257)	100.00	100.00	100.00	100.00	100.00	100 (0; 100-100)	
	Testing (n=1753)	99.70	99.80	99.90	99.90	100.00	99.86 (0.09; 99.4-100)	

#### COPD Classification Model

The model correctly predicted subgroup membership for 99.9% (14,443/14,457) of the cases in the full data set. Furthermore**,** as shown in Table 2, the internal validation results revealed that the percentage of COPD cases correctly assigned to a subgroup in the testing data set ranged from 99.1% to 100%, with a median (Q.50 as shown in Table 2) of 99.6%, and with 95% being in the range of 99.3% to 99.8%.

#### CHF Classification Model

The model correctly predicted the subgroup membership for 99.2% (25,476/25,672) of the cases in the full data set. Furthermore**,** as shown in Table 2, the internal validation results revealed that the percentage of CHF cases correctly assigned to a subgroup in the testing data set ranged from 98.7% to 99.7%, with a median (Q.50) of 99.3%, and with 95% being in the range between 99% to 99.6%.

#### THA/TKA Classification Model

The model correctly predicted subgroup membership in 100% (7010/7010) of the cases in the full data set. Furthermore**,** as shown in Table 2, the internal validation results revealed that the percentage of CHF cases correctly assigned to a subgroup in the testing data set ranged from 99.4% to 100%, with a median (Q.50) of 99.9%, and with 95% being in the range of 99.7% to 100%.

#### Application of the Classification Model to Generate Information for Other Models

The classification model was used to classify 100% of cases and 100% of controls for use in the prediction model (described in the next section). Furthermore, the proportion of cases and controls classified into each subgroup was used to calculate the risk of readmission for the respective subgroup (Multimedia Appendix 3). As this subgroup risk information was requested by the clinicians to improve the interpretability of the visual analytical model, the risk was juxtaposed next to the respective subgroups in the bipartite network visualizations (see blue text in Figures 2-4).

### Prediction Modeling

#### Overview

For each of the 3 index conditions, we developed 2 binary logistic regression models to predict readmission, with comorbidities in addition to sex, age, and race: (1) Standard Model representing all patients without subgroup membership, similar to the CMS models and (2) Hierarchical Model with an additional variable that adjusted for subgroup membership.

#### COPD Prediction Model

**Figure 5:**
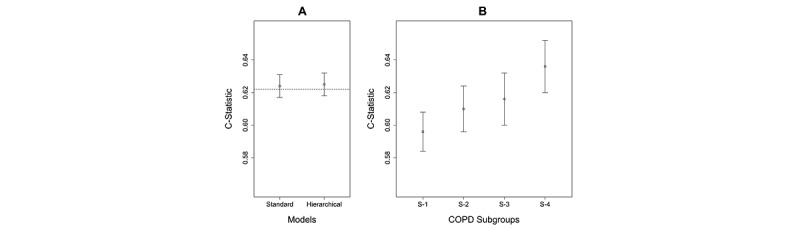
Predictive accuracy of the Standard Model compared with the Hierarchical Model in chronic obstructive pulmonary disease (COPD), as measured by the C-statistic. The C-statistic for the Centers for Medicare & Medicaid Services Standard Model is shown as a dotted line. (B) Predictive accuracy of the Standard Model when applied separately to patients classified to each subgroup. Subgroup-1 has lower accuracy than Subgroup-3 and Subgroup-4. (C-statistics in A and B cannot be compared, as they are based on models from different populations).

The inclusion and exclusion criteria (Multimedia Appendix 2) resulted in a cohort of 186,041 patients (29,026 cases and 157,015 controls). As shown in Figure 5A, the Standard Model had a C-statistic of 0.624 (95% CI 0.617-0.631) which was not significantly (*P*=.86) different from the Hierarchical Model that had a C-statistic of 0.625 (95% CI 0.618-0.632). The calibration plots revealed that both models had a slope close to 1 and an intercept close to 0 (Multimedia Appendix 5 [42-44]).

As shown in Figure 5B, the Standard Model was used to measure the predictive accuracy of patients in each subgroup. The results showed that Subgroup-1 had a lower C-statistic than Subgroup-3 and Subgroup-4. Although the C-statistics in Figures 5A and Figures 5B cannot be compared as they are based on models developed from different populations, these results reveal that the current CMS readmission model for CHF might be underperforming for a COPD patient subgroup, pinpointing which one might benefit from a Subgroup-Specific Model.

#### CHF Prediction Model

**Figure 6:**
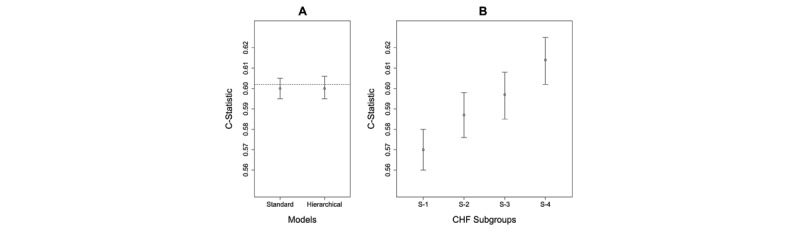
(A) Predictive accuracy of the Standard Model compared with the Hierarchical Model in congestive heart failure (CHF) as measured by the C-statistic. The C-statistic for the Centers for Medicare & Medicaid Services Standard Model is shown as a dotted line. (B) Predictive accuracy of the Standard Model when applied separately to patients classified to each subgroup. Subgroup-1 has lower accuracy than Subgroup-3 and Subgroup-4. (C-statistics in A and B cannot be compared, as they are based on models from different populations).

The inclusion and exclusion criteria (Multimedia Appendix 2) resulted in a cohort of 295,761 patients (51,573 cases and 244,188 controls). As shown in Figure 6A, the Standard Model had a C-statistic of 0.600 (95% CI 0.595-0.605), which was not significantly different (*P*=.29) from the Hierarchical Model, which also had a C-statistic of 0.600 (95% CI 0.595-0.606). The calibration plots revealed that all the models had a slope close to 1 and an intercept close to 0 (Multimedia Appendix 5).

As shown in Figure 6B, the Standard Model was used to measure the predictive accuracy of patients in each subgroup. The results showed that Subgroup-1 had a lower C-statistic than Subgroup-4. Although the C-statistics in Figures 6A and 6B cannot be compared as they are based on models developed from different populations, these results reveal that the current CMS readmission model for CHF might be underperforming for a CHF patient subgroup, pinpointing which one might benefit from a Subgroup-Specific model.

#### THA/TKA Prediction Model

**Figure 7:**
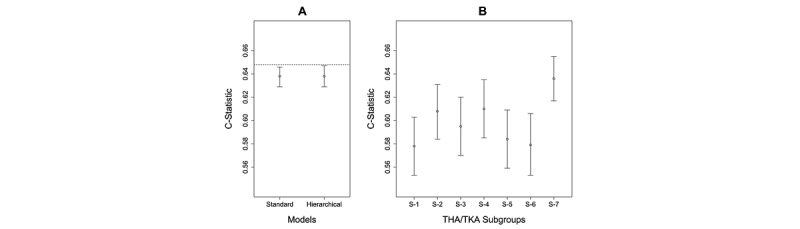
(A) Predictive accuracy of the Standard Model compared with the Hierarchical Model in total hip arthroplasty/total knee arthroplasty (THA/TKA) as measured by the C-statistic. The C-statistic for the Centers for Medicare & Medicaid Services Standard Model is shown as a dotted line. (B) Predictive accuracy of the Standard Model when applied separately to patients classified to each subgroup. Subgroup-1 has lower accuracy than Subgroup-7. (C-statistics in A and B cannot be compared, as they are based on models developed from different populations).

The inclusion and exclusion criteria (Multimedia Appendix 2) resulted in a cohort of 356,772 patients (16,520 cases and 340,252 controls). As shown in Figure 7A, the Standard Model had a C-statistic of 0.638 (95% CI 0.629-0.646), which was not significantly different (*P*=.69) from the Hierarchical Model, which had a C-statistic of 0.638 (95% CI 0.629-0.647). The calibration plots (Multimedia Appendix 5) revealed that both the models had a slope close to 1 and an intercept close to 0 (Multimedia Appendix 5).

As shown in Figure 7B, the Standard Model was used to measure the predictive accuracy of patients in each subgroup. The results showed that Subgroup-1 had a lower C-statistic than Subgroup-4. Again, although the C-statistics in Figures 7A and 7B cannot be compared as they are based on models developed from different populations, similar to the results in COPD, these results reveal that the current CMS readmission model for THA/TKA might be underperforming for 4 patient subgroups, pinpointing which ones might benefit from a Subgroup-Specific Model.

#### CMS Standard Model Versus CMS Hierarchical Model

Unlike the CMS published models, the models we developed used only the comorbidities that survived the feature selection. Therefore, to perform a head-to-head comparison with the published CMS models, we also developed a CMS Standard Model (using the same variables from the published CMS model) and compared it to the corresponding CMS Hierarchical Model (with an additional variable for subgroup membership) in each condition. Similar to the models in Figures 5-7, there were no significant differences in the C-statistics between the 2 modeling approaches in any condition (Multimedia Appendix 5). However, as shown in Table 3, the CMS Hierarchical Model for COPD had significantly higher NRI but not significantly higher IDI than the CMS Standard Model, whereas the CMS Hierarchical Model for CHF had a significantly lower NRI and IDI than the CMS Standard Model, and the CMS Hierarchical Model for THA/TKA had a significantly higher NRI but not significantly higher IDI than the CMS Standard Model. Furthermore, similar to the results presented in 6B, 7B, and 8B, when the CMS Standard Model was used to predict readmission separately in subgroups within each index condition, it identified subgroups that underperformed, pinpointing which ones might benefit from a Subgroup-Specific Model (Multimedia Appendix 5). In summary, the comparisons between the CMS Standard Models and the respective CMS Hierarchical Models showed that in the 2 conditions (COPD and THA/TKA), there was a small but statistically significant improvement in discriminating between the readmitted and not readmitted patients as measured by NRI, but not as measured by the C-statistic or IDI, and that a subgroup in each index condition might be underperforming when using the CMS Standard Model.

**Table 3:** Comparison of the Centers for Medicare & Medicaid Services (CMS) Standard Model with the CMS Hierarchical Model across the three index conditions based on net reclassification improvement (NRI) and integrated discrimination improvement (IDI).

Model	NRI												IDI				
	Categorical (95% CI)	*z* value		*P* value	Continuous (95% CI)		*z* value		*P* value			IDI (95% CI)			*z* value		*P* value
COPD^a^	0.023 (0.012 to 0.034)	−4.10	<.001		0.059 (0.034 to 0.083)	−4.68		<.001			0.0002 (−0.0004 to 0.0008)			−0.65		.51	
CHF^b^	−0.010 (−0.016 to −0.004)	3.27	.001		−0.038 (−0.057 to −0.019)	3.92				<.001	−0.0006 (−0.0009 to −0.0003)			3.92		<.001	
THA/TKA^c^	0.022 (0.012 to 0.032)	−4.31	<.001		0.111 (0.080 to 0.142)	−7.01				<.001	−0.003 (−0.004 to −0.002)			5.88		<.001	

^a^COPD: chronic obstructive pulmonary disease.

^b^CHF: congestive heart failure.

^c^THA/TKA: total hip arthroplasty/total knee arthroplasty.

## Discussion

### Overview

Our overall approach of using the MIPS framework to identify patient subgroups through visual analytics, and using those subgroups to build classification and prediction models revealed strengths and limitations for each modeling approach and for our data source. This examination provided insights for developing future clinical decision support systems and a methodological framework for improving the clinical interpretability of subgroup modeling results.

### Strengths and Limitations of Modeling Methods and Data Source

#### Visual Analytical Modeling

The results revealed three strengths of the visual analytical modeling: (1) the use of bipartite networks to simultaneously model patients and comorbidities enabled the automatic identification of patient-comorbidity biclusters and the integrated analysis of co-occurrence and risk; (2) the use of a bipartite modularity maximization algorithm to identify the biclusters enabled the measurement of the strength of the biclustering, critical for gauging its significance; and (3) the use of a graph representation enabled the results to be visualized through a network. Furthermore, the clinician stakeholders’ request to juxtapose the risk of each subgroup with their visualizations appeared to be driven by the need to reduce working memory loads (from having to remember that information when its spread over different outputs), which could have enhanced their ability to match bicluster patterns with chunks (previously learned patterns of information) stored in long-term memory. The resulting visualizations enabled them to recognize subtypes based on co-occurring comorbidities in each subgroup, reason about the processes that precipitate readmission based on the risk of each subtype relative to the other subtypes, and propose interventions that were targeted to those subtypes and their risks. Finally, the fact that the geriatrician could not fully interpret the THA/TKA network because it combined 2 fairly different conditions suggests that the clinical interpretations were not the result of a *confirmation bias* (interpretations leaning toward fitting the results).

However, the results also revealed two limitations: (1) although modularity is estimated using a closed-form equation (formula), no closed-form equation exists to estimate modularity variance, which is necessary to measure its significance. To estimate modularity variance, we used a permutation test by generating 1000 random permutations of the data and then compared the modularity generated from the real data, to the mean modularity generated from the permuted data. Given the size of our data sets (ranging from 7000 to 25,000 patients), this computationally expensive test took approximately 7 days to complete, despite the use of a dedicated server with multiple cores, and (2) although bicluster modularity was successful in identifying significant and meaningful patient-comorbidity biclusters, the visualizations themselves were extremely dense and therefore potentially concealed patterns within and between the subgroups. Future research should explore defining a closed-form equation to estimate modularity variance, with the goal of accelerating the estimation of modularity significance, and more powerful analytical and visualization methods to reveal intra- and intercluster associations in large and dense networks.

#### Classification Modeling

The results revealed two strengths of the classification modeling: (1) the use of a simple multinomial classifier was adequate to predict with high accuracy the subgroup to which a patient belonged; (2) because the model produced membership probabilities for each patient for each subgroup, the model captured the dense intercluster edges observed in the network visualization; and (3) the coefficients of the trained classifier could be inspected by an analyst, making it more transparent (relative to most deep learning classifiers that tend to be black boxes).

However, because we dichotomized the classification probabilities into a single subgroup membership, our approach did not fully leverage membership probabilities for modeling and visual interpretation. For example, some patients have high classification probabilities (representing strong membership) for a single subgroup (as shown by patients in the outer periphery of the biclusters with edges only within their bicluster), whereas others have equal probabilities for all subgroups (as shown in the inner periphery of the biclusters with edges going to multiple clusters). Future research should explore incorporating the probability of subgroup membership into the design of Hierarchical Models for improving predictive accuracy, and visualization methods for helping clinicians interpret patients with different profiles of membership strength, with the goal of designing patient-specific interventions.

#### Predictive Modeling

The results revealed two strengths of the predictive modeling: (1) the use of the Standard Model to measure predictive accuracy across the subgroups helped to pinpoint which subgroups tended to have lower predictive accuracy than the rest and therefore which of them could benefit from a more complex but accurate Subgroup-Specific Model and (2) despite the use of a simple Hierarchical Model with a dichotomized membership label for each patient, the predictive CMS models detected significant differences in the prediction accuracy as measured by NRI in 2 of the conditions, when compared with the CMS Standard Models. However, the results also revealed that the differences in predictive accuracy as measured by the C-statistic and NRI were small, suggesting that comorbidities alone were potentially insufficient for accurately predicting readmission. Future research should explore the use of electronic health records and multiple subgroup-specific models targeted to each subgroup (enabling each model to have different slopes and intercepts) to potentially improve the predictive accuracy of the prediction models.

#### Data Source

The Medicare claims data had four key strengths: (1) the scale of the data sets that enabled subgroup identification with sufficient statistical power; (2) spread of the data collected from across the United States, which enabled generalizability of the results; (3) data about older adults, which enabled examination of subgroups in an underrepresented segment of the US population; and (4) data used by CMS to build predictive readmission models, which enabled a head-to-head comparison with the Hierarchical Modeling approach.

However, these data had two critical limitations: (1) as we compared our models with the CMS models, we had to use the same definition for controls (90 days with no readmission) that had been used, which introduced a selection bias that exaggerated the separation between cases and controls. Similarly, by excluding patients who died, this exclusion criterion potentially biased the results toward healthier patients and (2) administrative data have known limitations, such as the lack of comorbidity severity and test results, which could strongly impact the accuracy of predictive models. Future research should consider the use of national-level electronic health record data, such as those assembled by the National COVID Cohort Collaborative [54] and the TriNetX [55] initiatives, which could overcome these limitations by providing laboratory values and comorbidity severity but could also introduce new as yet unknown limitations.

### Implications for Clinical Decision Support That Leverage Patient Subgroups

Although the focus of this project was to develop and evaluate the MIPS framework, its application to 3 index conditions, coupled with extensive discussions with clinicians, led to insights for designing a future clinical decision support system. Such a system could integrate the outputs from all 3 models in MIPS. As we have shown, the visual analytical model automatically identified and visualized the patient subgroups, which enabled the clinicians to comprehend the co-occurrence and risk information in the visualization, reason about the processes that lead to readmission in each subgroup, and design targeted interventions. The classification model leveraged the observation that many patients have comorbidities in other biclusters (shown by a large number of edges between biclusters) and accordingly generated a membership probability (MP) of a patient belonging to each bicluster, from which the highest was chosen for bicluster membership. Finally, the predictive model calculated the risk of readmission for a patient by using the most accurate model designed for the bicluster to which the patient belonged.

The outputs from these models could be integrated into a clinical decision support system to provide recommendations for a specific patient using the following algorithm: (1) use the classifier to generate the MP of a new patient belonging to each subgroup; (2) use the predictive model to calculate the risk (R) of that patient in each subgroup; (3) generate an importance score (IS) for each subgroup, such as by calculating a *membership-weighted risk* [MP x R]; (4) rank the subgroups and their respective interventions using IS; and (5) use the ranking to display in descending order, the subgroup comorbidity profiles along with their respective potential mechanisms, recommended treatments, and the respective IS. Such model-based information, displayed through a user-friendly interface, could enable a clinician to rapidly scan the ranked list to (1) determine why a specific patient profile fits into one or more subgroups, (2) review the potential mechanisms and interventions ranked by their importance, and (3) use the combined information to design a treatment that is customized for the real-world context of the patient. Consequently, such a clinical decision-support system could not only provide a quantitative ranking of membership to different subgroups and the IS for the associated interventions, but also enable the clinician to understand the rationale underlying those recommendations, making the system interpretable and explainable. Our current work explores a framework called Model-based Subtype and Treatment Recommendations (MASTR) for developing such clinical decision-support systems, and evaluating them to determine their clinical efficacy in comparison to standard-of-care.

### Implications for Analytical Granularity to Enhance the Interpretability of Patient Subgroups

Although the visual analytical model enabled clinicians to interpret the patient subgroups, they were unable to interpret the associations within and between the subgroups because of the large number of nodes in each bicluster and the dense edges between them. Several network filtering methods [56,57] have been developed to *thin out* such dense networks such as by dropping or bundling nodes and edges based on user-defined criteria, to improve visual interpretation. However, such filtering could bias the results or modify the clusters resulting from reduced data.

An alternate approach that preserves the full data set leverages the notion of analytic granularity, in which the data are progressively analyzed at different levels. For example, we have analyzed patients with COVID-19 [11] at the cohort, subgroup, and patient levels, and we are currently using the same approach to examine symptom co-occurrence and risk at each level in patients with Long COVID. Our preliminary results suggest that analyzing data at different levels of granularity enables clinicians to progressively interpret patterns, such as within and between subgroups, in addition to guiding the systematic development of new algorithms. For example, at the subgroup level, we have designed an algorithm that identifies which patient subgroups have a significantly higher probability of having characteristics that are clustered in another subgroup, providing critical information to clinicians about how to design interventions for such overlapping subgroups. Furthermore, at the patient level, we have identified patients that are very dissimilar to their subgroups based on their pattern of characteristics inside and outside their subgroup. Such dissimilar patients could be flagged to examine whether they need individualized interventions compared with those recommended for the rest of their subgroups. Such analytical granularity could therefore inform the design of interventions by clinicians in addition to the design of decision support systems that provide targeted and interpretable recommendations to physicians, who can then customize them to fit the real-world context of a patient.

### Implications of the MIPS Framework for Precision Medicine

Although we have demonstrated the application of the MIPS framework across multiple readmission conditions, its architecture has 3 properties that should enable its generalizability across other medical conditions. First, as shown in Figure 1, the framework is *modular* with explicit inputs and outputs, enabling the use of other methods in each of the 3 modeling steps. For example, the framework can use other biclustering (eg, nonnegative matrix factorization) [58], classification (eg, deep learning) [59], and prediction methods (eg, subgroup-specific modeling) [16]. Second, the framework is *extensible,* enabling elaboration of the methods at each modeling step to improve the analysis and interpretation of subgroups. For example, as discussed earlier, analytical granularity at the cohort, subgroup, and patient levels could improve the interpretability of subgroups in large and dense data sets. Third, the framework is *integrative* as it systematically combines the strengths of machine learning and statistical and precision medicine approaches. For example, visual analytical modeling leverages search algorithms to discover co-occurrence in large data sets, classification and prediction modeling leverages probability theory to measure the risk of co-occurrence patterns, and clinicians leverage medical knowledge and human cognition to interpret patterns of co-occurrence and risk for designing precision medicine interventions. Therefore, the integration of these different models with a focus on their clinical interpretation operationalizes *team-centered informatics* [60] designed to facilitate data scientists, biostatisticians, and clinicians in multidisciplinary translational teams [61] to work more effectively across disciplinary boundaries with the goal of designing precision medicine interventions. Our current research tests the generality of the MIPS framework in other conditions, such as in Long COVID and poststroke depression, with the goal of designing and evaluating precision medicine interventions targeted to patient subgroups.

### Conclusions

Although several studies have identified patient subgroups in different health conditions, there is a considerable gap between the identification of subgroups and their modeling and interpretation for clinical applications. Here, we developed MIPS, a novel analytical framework to bridge this gap, using a 3-step modeling approach. A visual analytical method automatically identified statistically significant and replicated patient subgroups and their frequently co-occurring comorbidities, which were clinically significant. Next, a multinomial logistic regression classifier was highly accurate in correctly classifying patients into subgroups identified by the visual analytical model. Finally, despite using a simple hierarchical logistic regression model to incorporate subgroup information, the predictive models showed a statistically significant improvement in discriminating between readmitted and not readmitted patients in 2 of the 3 readmission conditions, and additional analysis pinpointed for which patient subgroups the current CMS model might be underperforming. Furthermore, the integration of the 3 models helped to (1) elucidate the data input and output dependencies among the models, enabling clinicians to interpret the patient subgroups, reason about mechanisms precipitating hospital readmission, and design targeted interventions and (2) provide a generalizable framework for the development of future clinical decision support systems that integrate outputs from each of the 3 modeling approaches.

However, the evaluation of MIPS across the 3 readmission index conditions also helped to identify the limitations of each modeling method, and of the data. The visual analytical model was too dense to enable clinicians to interpret the associations within and between subgroups, and the absence of a closed-form equation to measure modularity variance required a computationally expensive process to measure the significance of the biclustering. Furthermore, the small improvement in predictive accuracy suggested that comorbidities alone were insufficient for accurately predicting hospital readmission.

By leveraging the modular and extensible nature of the MIPS framework, future research should address these limitations by developing more powerful algorithms that analyze subgroups at different levels of granularity to improve the interpretability of intra- and intercluster associations and the evaluation of subgroup-specific models to predict outcomes. Furthermore, data from electronic health records made available through national-level data initiatives, such as National COVID Cohort Collaborative and TriNetX, now provide access to critical variables, including laboratory results and comorbidity severity, which should lead to higher accuracy in predicting adverse outcomes. Finally, extensive discussions with clinicians have confirmed the need for decision support systems that integrate outputs from the 3 models to provide for a specific patient, predicted subgroup memberships, and ranked interventions, along with associated subgroup profiles and mechanisms. Such interpretable and explainable systems could enable clinicians to use patient subgroup information for informing the design of precision medicine interventions, with the goal of reducing adverse outcomes such as unplanned hospital readmissions and beyond.

## Appendix

Multimedia Appendix 1 Analytical methods for modeling and interpreting patient subgroups.

Multimedia Appendix 2 Patient inclusion and exclusion criteria.

Multimedia Appendix 3 Variable and feature selection.

Multimedia Appendix 4 Classification modeling.

Multimedia Appendix 5 Predictive modeling.

## Abbreviations

CHF: congestive heart failure
CMS: Centers for Medicare & Medicaid Services
COPD: chronic obstructive pulmonary disease
IDI: integrated discrimination improvement
IS: importance score
MIPS: modeling and interpreting patient subgroups
MP: membership probability
NRI: net reclassification improvement
OR: odds ratio
RI: Rand Index
THA/TKA: total hip arthroplasty/total knee arthroplasty
