# Supplementary material for: A Framework for Modeling and Interpreting Patient Subgroups Applied to Hospital Readmission: Visual Analytical Approach
Source: JMIR Med Inform. 2022 Dec 7;10(12):e37239. doi: 10.2196/37239 (PMC9773032; doi:10.2196/37239)
Supplement: Multimedia Appendix 1 [file medinform_v10i12e37239_app1.docx]

## APPENDIX-1

## Analytical Methods for the MIPS Framework

| Model | Inputs | Outputs |
| --- | --- | --- |
| 1. **Visual Analytical Model** (Bipartite Network Analysis) | - **Training Dataset**: 50% random sample of 100% cases, and an equal number of 1:1 matched controls (used only for feature selection) - **Replication Dataset**: 50% random sample of 100% cases and equal number of 1:1 matched controls | - **Model Training**   Feature Selection: Set of comorbidities univariably significant in both the training and replication datasets  Biclustering: Modularity (degree of biclusteredness) and its significance, number of biclusters (subgroups), and their patient and comorbidity members in the training and replication datasets   - **Model Replication**   Comorbidity Co-Occurrence: Rand Index (degree of replication), and its significance to measure replicability of comorbidity co-occurrence   - **Model Interpretation**   Visualization: Layout of the bipartite network juxtaposed with risk of individual comorbidities and subgroups  Clinical Significance: Interpretation by clinicians for face validity of patient subgroups based on comorbidity co-occurrence, leading to inference of mechanisms precipitating readmission, and interventions |
| **2. Classification** **Model** (Multinomial Logistic Regression) | - **Training Dataset**: Random sample of 75% cases, with bicluster membership - **Internal Validation Dataset**: Random sample of 25% of cases (with subgroup membership used to validate the model) | - **Model Training**   Subgroup Membership: Probability of membership of each case to each subgroup (soft labels), with the highest used to determine subgroup membership (hard labels)   - **Model Internal Validation**   Internal Validation: Accuracy of classification model based on hard labels   - **Model Application**   Classification: Subgroup classification of 100% cases and 100% controls  Subgroup Risk: Proportion of cases in each subgroup |
| 3. **Prediction Model** (Binary Logistic Regression, and Hierarchical Binary Logistic Regression) | - **Training Dataset**: Random samples of 75% of 100% cases and controls, with subgroup membership - **Internal Validation Dataset**: Random sample of 25% of cases and controls (with case/control labels used to validate the model) | - **Model Training**   Predicted Risk: Each patient’s probability of being readmitted.   - **Model Internal Validation**   Internal Validation: C-statistic (discrimination), and calibration-in-the-large and calibration slope (calibration)   - **Model Comparison**   Accuracy: Net Reclassification Improvement (NRI) and Integrated Discrimination Improvement (IDI) |

**Table 1**. Inputs used to train and replicate/validate the three models, and the analytical outputs they produced.

*Visual Analytical Modeling.* The data used to build the visual analytical model consisted of 100% cases, and an equal number of 1:1 matched controls extracted by randomly selecting a control without replacement to match each case based on age, gender, race/ethnicity, and Medicaid eligibility [45]. The resulting dataset was divided randomly into a training (50%) and replication (50%) dataset (we use the term *replication* to avoid confusion with the term *validation* typically used in classification and prediction models). We used a bipartite network to model the cases (30-day readmitted patients) and significant comorbidities in each index condition using the following steps:

1. *Model Training.* The training of the bicluster network model consisted of the following two steps:
2. *Feature Selection.* Given the large number of patients and comorbidities in the dataset, we used feature selection to identify comorbidities with the strongest signal and therefore interpretability for readmission using the following steps: (1) excluded comorbidities with prevalence less than 1% (as is commonly done in studies to reduce noise [23]); (2) selected significant comorbidities in the training dataset based on a 2-way interaction test using odds ratio (OR) with directionality, and correcting for multiple testing using Bonferroni, and (3) tested the surviving comorbidities for replication in the replication dataset, and selected those that were significant in both datasets. Appendix-2 shows the number of comorbidities, and variables that were included in the analysis for each of the three index conditions. The above feature selection generated a single set of significant and replicated comorbidities used for the following bipartite network analysis.
3. *Biclustering.* We used bipartite networks analysis [25] on the training dataset to analyze heterogeneity in readmission using the following steps: (1) Removed all cases that did not have any comorbidities (as the modularity maximization algorithm will trivially put disconnected nodes into a separate cluster). (2) Represented the cases (30-day readmitted patients in the training dataset) and their significant and replicated comorbidities (selected in Step A) as a bipartite network. As shown in Fig. 1, the nodes represented cases (circles) or comorbidities (triangles), and edges (lines) represented which case had which comorbidity. (3) Used a bipartite modularity maximization algorithm [26-28], to identify the number of biclusters, their members, and degree of biclusteredness of the network using modularity. Modularity is defined as the fraction of edges falling within a cluster, minus the expected fraction of such edges in a network of the same size with randomly assigned edges. Modularity ranges from -0.5 to +1, with values >0 indicating biclustering that is higher than can be expected by chance. We used the bipartite version of modularity to find biclusters in the network. (4) Measured the significance of the bicluster modularity by comparing it to a distribution of the same quantity generated from 1000 random permutations of the network, by preserving the network size (number of nodes) and the network density (number of edges).


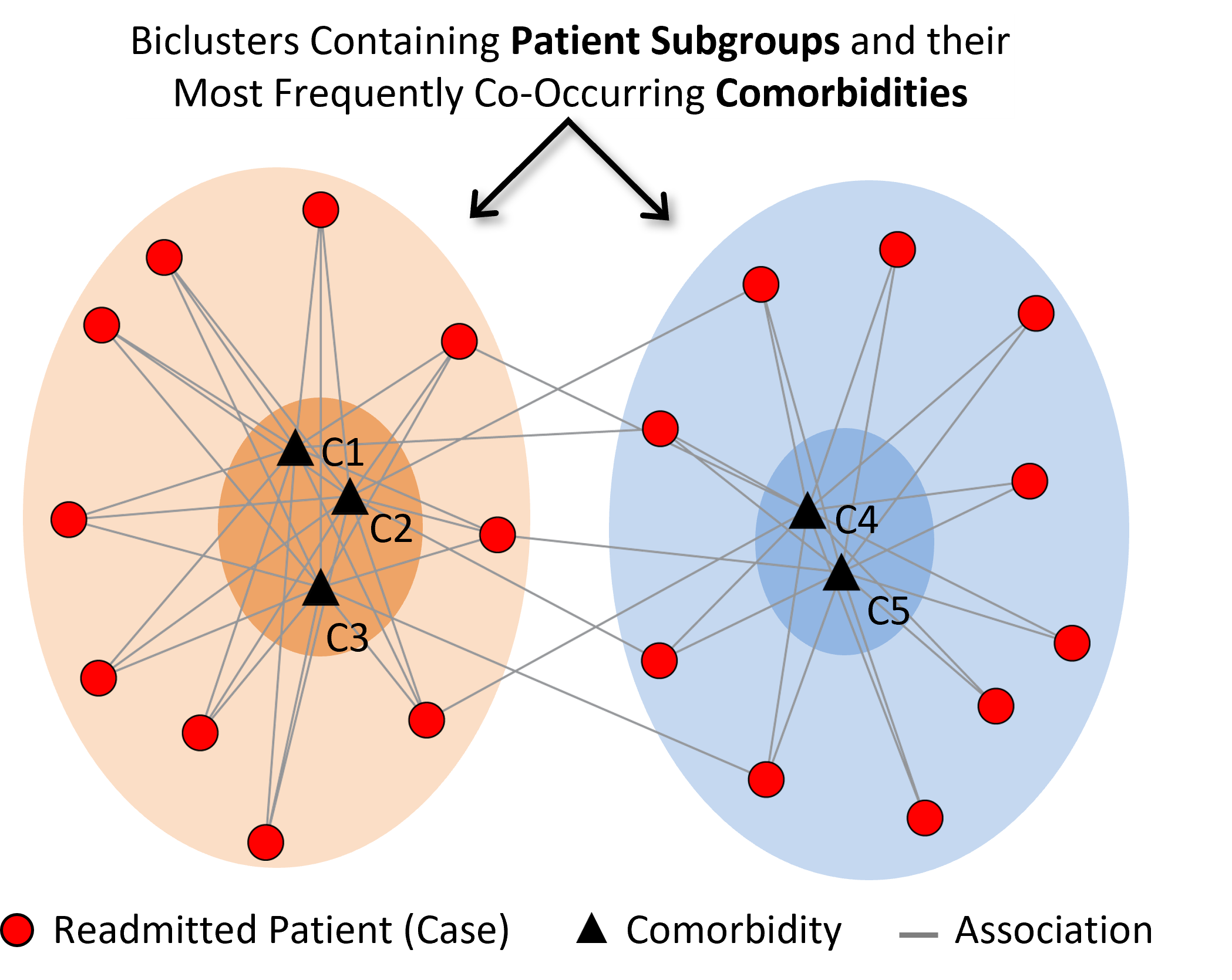


**Fig. 1.** A bipartite network showing patient subgroups and their most frequently co-occurring comorbidities.

1. *Model Replication.* Repeated the above biclustering steps 1-4 to identify subgroups in the replication dataset, and compared the comorbidity co-occurrence in the training dataset, to that in the replication dataset using the Rand index (RI) [46]. RI measures the proportion of comorbidity pairs that co-occurred and did not co-occur in a cluster in the training and replication datasets (where 0=no inter-network cluster similarity, and 1=total inter-network cluster similarity). The significance of RI was measured by comparing it to a distribution of the same quantity generated from 1000 random permutations of the training and replication networks. All tests of statistical significance in Steps A and B were 2-sided.
2. *Model Interpretation.* The model interpretation consisted of the following steps:
3. *Visualization.* We used the following steps to visualize the network generated from the training dataset. (1) Used *Fruchterman-Reingold* (FR) [29], a force-directed algorithm to lay out the bipartite network. This layout algorithm pulls together nodes that are strongly connected, and pushes apart nodes that are not. This results in nodes with a similar pattern of connections to be placed close to each other in Euclidean space, and those that are dissimilar are pushed apart. (2) As the FR algorithm often cannot entirely separate clusters in large and dense networks, the network layout needs to be visually enhanced before it is interpretable by clinician stakeholders. Therefore, we used the *ExplodeLayout* algorithm [30, 31] to separate the biclusters to reduce their visual overlap. This algorithm preserves the distances of nodes within a bicluster, but increases the distance of nodes between clusters to improve interpretability. (3) Juxtaposed the risk of readmission with the network visualization (in response to a request from the clinical stakeholders). This was done by (a) displaying comorbidity labels with their univariable ORs for readmission (measured in Step A) ranked by their odds ratios (ORs) for each subgroup, and (b) measuring the readmission risk for each patient subgroup based on the full case-control population (explained in more detail in the section on classification modeling), and juxtaposing it with the respective subgroup.
4. *Clinical Interpretation.* We used the following steps to solicit clinical interpretations of the above bipartite network. (1) Recruited a pulmonologist specializing in COPD and hospital readmission to interpret the COPD results, and a geriatrician with expertise in treating older adults in CHF and THA/TKA to interpret the respective results. (2) Requested each clinician stakeholder to interpret the patient subgroups, their mechanisms, and potential interventions to reduce the risk for readmission.

*Classification Modeling.* As shown in the bipartite network example in Fig. 1, the biclusters identified through the modularity maximization algorithm contain patient subgroups and their *most frequently* co-occurring comorbidities with respect to other patients in the network. However, there are often many edges between biclusters, revealing that many patients within a bicluster have comorbidities that exist in other biclusters. As is true for most partitioning cluster methods, including modularity, membership of a new patient to each bicluster is therefore *probabilistic*. The classification of a patient into a cluster is therefore not defined by the *inclusion or exclusion of comorbidities* (e.g., hypertension and diabetes), but rather by the *probability* of being in a patient subgroup. Patients are therefore similar or different, not just in a handful of carefully-selected comorbidities while ignoring others, but based on *all* of their recorded comorbidities. This overall profile of patients reflects the reality of comorbid conditions.

To model the above complexity, we used multinomial logistic regression [16] to develop classification models in each index condition. This approach has the advantage of generating probabilities (“soft labels”) for a patient to belong to each patient subgroup. The models were trained, internally validated, and then applied to generate information for the other two modeling methods, as described below:

1. *Model Training.* The data used to build the classification model consisted of the training dataset and subgroup membership from the visual analytical model. We trained a multinomial logistic regression model using the above data, with independent variables that included comorbidities identified through feature selection done for the visual analytical modeling. Accuracy of the trained model was measured by calculating the percentage of times the model correctly classifed the cases into the subgroups, using the highest predicted probability across the subgroups (“hard labels”).
2. *Model Internal Validation.* To internally validate the classifier, we randomly split the above data into training (75%) and testing (25%) datasets, 1000 times. For each iteration, we trained a model using the training dataset, and measured its accuracy on the testing dataset. This was done by predicting the subgroup membership using the highest predicted probability among all the subgroups. The overall predicted accuracy was then estimated by calculating the mean accuracy across the 1000 models.
3. *Model Application.* Using the 100% cases, in addition to the 100% controls from July 2013-August 2014 (representing the entire Medicare population of each index condtion from those years), we generated the following two types of information for use in the other models. (1) Used the classifier trained in Step A above, to classify 100% cases and 100% controls into a subgroup. This information was used by the subsequent predictive modeling. (2) While the visual analytical model used the 1:1 matched controls for feature selection, this cohort did not represent the entire population. Therefore, to accurately measure the subgroup risk, we used the entire case-control population classified into the subgroups (as described in the above step), and measured the proportion of cases in each subgroup. Furthermore, as requested by the clinicians, we juxtaposed these subgroup risks next to the respective subgroups in the bipartite network visualization, to improve their interpretability.

*Predictive Modeling.* The data used to build the predictive models consisted of 100% cases and 100% controls, in addition to their subgroup membership generated from the above classification models. These data were randomly spilt into a training (75%) and validation (25%) dataset. The predictive models were trained, internally validated, and compared for predictive accuracy, as described below:

1. *Model Training.* We used the training dataset to train a Standard Model (binary logistic regression without subgroup membership similar to the CMS models), and a Hierarchical Model (binary logistic regression with subgroup membership), with 30-day unplanned readmission (yes vs. no) as the outcome. Independent variables for both models included comorbidities identified through the feature selection in each index condition (see Appendix-2), and demographics. The Hierarchical Model additionally included subgroup membership.
2. *Model Internal Validation.* We used the validation dataset to internally validate the models through the following two measures:
3. Discrimination (model’s ability to distinguish readmitted patients from those not readmitted) was measured using the C-statistic, which is identical to the area under the receiver operating characteristic (ROC) curve. Model discrimination was examined using box plots to show the average risk prediction for patients with and without readmission.
4. Calibration (model’s agreement of the predicted probabilities with the observed risk) was measured using calibration-in-the-large, and calibration slope, which was examined through a calibration plot showing the proportion of patients actually admitted, versus deciles of predicted probability of having readmission. Good calibration is when calibration-in-the-large is close to zero, and the calibration slope is close to one. Since the large sample size overpowered the study, we did not measure the calibration based on statistical significance (e.g., *P* values of the Hosmer-Lemeshow and calibration indices).
5. *Model Comparisons.* We used the chi-squared test to compare the C-statistic of the Standard Model to that of the Hierarchical Model. We also measured the C-statistic of the Standard Model applied to each subgroup separately. This enabled examination of how the Standard Model performed on patient subgroups to identify, for example, which subgroups underperformed when using the current Standard Model.

Because the above models used the feature selection step to select comorbidities for use as independent variables, they differed from those used in the published CMS models. Therefore, to perform a head-to-head comparison with the published CMS models, we additionally developed a logistic regression model using independent variables that were identical to the published CMS model (CMS Standard Model), which was compared to the same model that included subgroup membership (CMS Hierarchical Model). We used the chi-squared test to compare the C-statistic of the CMS Standard Model to that from the CMS Hierarchical Model, in addition to the following measures of model accuracy:

1. Net Reclassification Improvement (NRI) measured the proportion of patients whose predicted probability of readmission improved with reference to actual readmission status. We used two NRI statistics: (a) categorical NRI, which predicted readmission probabilities divided into 10 sequential categories ranging from 0-1, with improvement requiring a shift between categories; and (b) continuous NRI which is based on the proportions of patients with any improved predicted probability of readmission, regardless of the size of that improvement.
2. Integrated Discrimination Improvement (IDI) measured the difference in the average improvement in predicted risks between the CMS Standard Model and the CMS Hierarchical Model.
