# Supplementary material for: A Framework for Modeling and Interpreting Patient Subgroups Applied to Hospital Readmission: Visual Analytical Approach
Source: JMIR Med Inform. 2022 Dec 7;10(12):e37239. doi: 10.2196/37239 (PMC9773032; doi:10.2196/37239)
Supplement: Multimedia Appendix 2 [file medinform_v10i12e37239_app2.docx]

## APPENDIX-2

## Patient Inclusion and Exclusion Criteria

**ICD-9 Codes for Selecting Index Conditions**

1. **Hospitalization for COPD** was defined as (1) hospitalization with a primary ICD-9 code for COPD (491.21, 491.22, 491.8, 491.9, 492.8, 493.20, 493.21, 493.22 and 496), or (2) primary ICD-9 codes of 518.81, 518.82, 518.84 or 799.1 and secondary ICD-9 codes for COPD (491.21, 491.22, 493.21 and 493.22).
2. **Hospitalization for CHF** was defined as hospitalization with a primary ICD-9 code for CHF (402.01, 402.01, 402.91, 404.01, 404.03, 404.11, 404.13, 404.91, 404.93 or 428.xx)
3. **Hospitalization for THA/TKA** was defined as hospitalization with a primary ICD-9 code for THA/TKA (81.51 and 81.54). We included admissions only for elective total hip/knee arthroplasty, and those with non-elective were excluded. These included admissions with a diagnosis of a femur, hip, or pelvic fracture, those who received partial hip arthroplasty, revision or resurfacing procedures concurrently with hip/knee arthroplasty, those with malignant bone neoplasm, or with a procedure code for removal of implanted devices/prostheses.

**Inclusion and Exclusion Criteria**

For each index condition, we used the same inclusion and exclusion criteria used to develop the CMS models, but with the most recent years (2013-2014) provided by Medicare when we started the project. We used 100% of the 30-day readmitted patients in 2013 and 2014 Medicare claims data, from which we extracted all patients that were admitted to an acute care hospital on or after July 2013-August 2014 with a principal diagnosis of the index condition, were 66 years of age or older, and were enrolled in both Medicare parts A and B fee-for-service plans in the 6 months before admission. Furthermore, we excluded patients who were transferred from other facilities, died during the hospitalization, or transferred to another acute care hospital. Similar to the CMS models, we selected the first admission for patients with multiple admissions during the study period, and did not use Medicare Part D (related to prescription medications).

Next, we extracted 100% controls who were not readmitted for at least 90 days since discharge. CMS uses this 90-day window of no re-admittance to ensure that the controls are substantially free of complications that result in readmission during this period [44]. A small percentage (0.8%) of Medicare patients had “unknown race” for the Race attribute, so we grouped “unknown race” and “other race” and ensured that there was an equal number of them in the cases and control datasets. The low rate of missing data on race had too low a risk for bias to warrant a sensitivity analysis. The following flow charts describe the inclusion and exclusion criteria used to extract cases and controls for COPD, CHF, and THA/TKA, and the respective numbers of patients extracted at each step.

**Patient Inclusion and Exclusion Criteria for COPD Training and Replication Datasets**

**
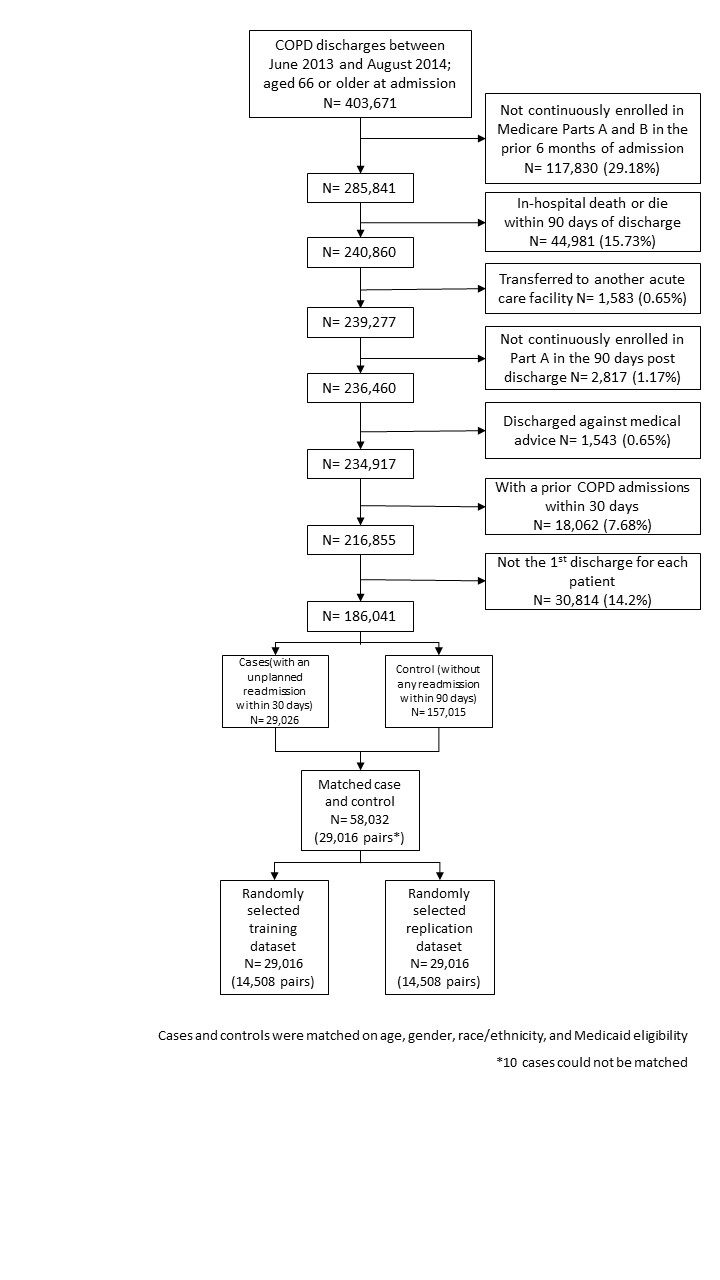
**

July

**Patient Inclusion and Exclusion Criteria for CHF Training and Replication Datasets**

July

**Patient Inclusion and Exclusion Criteria for THA/TKA Training and Replication Datasets**

July
