# Supplementary material for: A Framework for Modeling and Interpreting Patient Subgroups Applied to Hospital Readmission: Visual Analytical Approach
Source: JMIR Med Inform. 2022 Dec 7;10(12):e37239. doi: 10.2196/37239 (PMC9773032; doi:10.2196/37239)
Supplement: Multimedia Appendix 3 [file medinform_v10i12e37239_app3.docx]

## APPENDIX-3

## Variable and Feature Selection

**COPD**

The initial set of comorbidities included 45 comorbidities generated from a union of the three comorbidity indices, plus 2 condition-specific comorbidities recommended by the clinicians, resulting in 47 comorbidities. The following feature-selection steps resulted in 30 comorbidities surviving, that were used for the modeling:

1. Removed comorbidities with prevalence less than 1%, resulting in the following that were excluded, leaving 44 comorbidities:

| **Excluded Comorbidities** | **Label** |
| --- | --- |
| 1. RespDepend | Respirator dependence/respiratory failure (V22 CC 82-83) |
| 1. Pancreatitis | Chronic pancreatitis (V22 CC 34) |
| 1. HIV_AIDS | HIV/AIDS |

1. Measured the OR of each comorbidity for readmission and excluded the following that were not significant (at the .05 level corrected for multiple testing with Bonferroni), leaving 40 comorbidities:

| **Excluded Comorbidities** | **Label** |
| --- | --- |
| 1. Neoplasm_other | Other digestive and urinary neoplasms (V22 CC 14) |
| 1. Lymphatic | Lymphatic, head and neck, brain, and other major cancers; breast, colorectal and other cancers and tumors; other respiratory and heart neoplasms |
| 1. Diabetes_wo_comp | Diabetes without complications |
| 1. Rheumatic | Connective Tissue Disease-Rheumatic Disease |

1. Conducted a two-way co-occurrence test resulting in none being excluded.
2. Conducted a two-way directionality test resulting in the following that were excluded:

| **Excluded Comorbidities** | **Label** |
| --- | --- |
| 1. Anxiety | Anxiety disorders (V22 CC 62) |
| 1. Brain_disorder | Dementia or other specified brain disorders |
| 1. Liver_disease | Liver disease |
| 1. LungCa | Lung and other severe cancers (V22 CC 9) |
| 1. Metastatic_cancer | Metastatic cancer or acute leukemia |
| 1. Vertebral_fract | Vertebral fractures without spinal cord injury (V22 CC 169) |

1. Repeated steps 2-4 in the replication dataset resulting in 30 comorbidities shown below:

| **Final Comorbidities** | **Label** |
| --- | --- |
| 1. Anemias | Iron deficiency or other/unspecified anemias and blood disease |
| 1. Apnea | Sleep apnea |
| 1. Arrhythmias | Specified arrhythmias and other heart rhythm disorders |
| 1. CardioRespShock | Cardio-respiratory failure and shock (V22 CC 84) |
| 1. Cellulitis | Cellulitis, local skin infection (V22 CC 164) |
| 1. Coronary_angina | Coronary atherosclerosis or angina (V22 CC 88-89) |
| 1. Coronary_syndrome | Acute coronary syndrome |
| 1. Depression | Depression (V22 CC 61) |
| 1. Diabetes_w_comp | Diabetes with complications |
| 1. Endocrine_disorder | Endocrine and metabolic disorders; disorders of fluid/electrolyte/acidbase balance |
| 1. GI_other | Other gastrointestinal disorders (V22 CC 38) |
| 1. HD_other | Other and unspecified heart disease (V22 CC 98) |
| 1. Heart_failure | Congestive heart failure |
| 1. Hemiplegia | Hemiplegia, paraplegia, paralysis, functional disability |
| 1. Hypertension_comp | Hypertension complicated |
| 1. Hypertension_Uncomp | Hypertension uncomplicated |
| 1. Infection | Severe infection; other infectious diseases (V22 CC 3-7) |
| 1. Malnutrition | Protein-calorie malnutrition / weight loss |
| 1. Morbid_OB | Morbid obesity; other endocrine/metabolic/nutritional disorders |
| 1. MV | History of mechanical ventilation |
| 1. Neurological_Disorders | Other Neurological Disorders |
| 1. Neuropathy | Polyneuropathy / other neuropathies (V22 CC 75,81) |
| 1. Peptic_ulcer | Peptic ulcer, hemorrhage, other specified gastrointestinal disorders |
| 1. Pneu | Pneumonia (V22 CC 114-116) |
| 1. psych_other | Other psychiatric disorders (V22 CC 63) |
| 1. Psychosis | Drug/alcohol psychosis or dependence |
| 1. Renal_failure | Renal failure |
| 1. Ulcer | Decubitus ulcer or chronic skin ulcer (V22 CC 157-161) |
| 1. Valvular_Disease | Valvular Disease |
| 1. Vascular | Vascular or circulatory disease |

**CHF**

The initial set of comorbidities included 42 comorbidities generated from a union of the three comorbidity indices, plus 1 condition-specific comorbidities recommended by the clinicians, resulting in 43 comorbidities. The following feature-selection steps resulted in 37 comorbidities, that were used for the modeling:

1. Removed comorbidities with prevalence less than 1%, resulting in the following that were excluded, leaving 42 comorbidities:

| **Excluded Comorbidities** | **Label** |
| --- | --- |
| 1. HIV_AIDS | HIV/AIDS |

1. Measured the OR of each comorbidity for readmission and excluded the following that were not significant (at the .05 level corrected for multiple testing with Bonferroni), leaving 40 comorbidities that had significant associations with readmission:

| **Excluded Comorbidities** | **Label** |
| --- | --- |
| 1. Metastatic_cancer | Metastatic cancer or acute leukemia |
| 1. Diabetes_wo_comp | Diabetes Mellitus without Complication |

1. Conducted a two-way co-occurrence test resulting in none being excluded.
2. Conducted a two-way directionality test, resulting in the following that were excluded leaving 39 variables that were involved in one or more significant direction tests:

| **Excluded Comorbidities** | **Label** |
| --- | --- |
| 1. Rheumatic | Rheumatic Disease |

1. Repeated steps 2-4 in the replication dataset resulting in 37 comorbidities shown below:

| **Final Comorbidities** | **Label** |
| --- | --- |
| 1. Coronary_angina | Coronary atherosclerosis or angina (CC 88-89) |
| 1. CABG | History of coronary artery bypass graft (CABG) surgery |
| 1. COPD | Chronic Obstructive Pulmonary Disease (CC 111) |
| 1. CardioRespShock | Cardio-respiratory failure and shock |
| 1. Depression | Depression (CC 61) |
| 1. Dialysis | Dialysis status (CC 134) |
| 1. GI_other | Other gastrointestinal disorders (CC 38) |
| 1. Hypertension_Comp | Hypertension Complicated |
| 1. Hypertension_Uncomp | Hypertension Uncomplicated |
| 1. Hypothyroidism | Hypothyroidism |
| 1. Nephritis | Nephritis (CC 141) |
| 1. Obesity | Obesity |
| 1. Neuro_disorders | Other Neurological Disorders |
| 1. HD_other | Other and unspecified heart disease (CC 98) |
| 1. Psych_other | Other psychiatric disorders (CC 63) |
| 1. Urinary_tract_disorder | Other urinary tract disorders (CC 145) |
| 1. Pneu | Pneumonia (CC 114-116) |
| 1. Renal_failure | Renal failure (CC 135-140) |
| 1. Ulcer | Decubitus ulcer or chronic skin ulcer (CC 157-161) |
| 1. Coronary_syndrome | Acute coronary syndrome |
| 1. Psychosis | Drug/alcohol abuse/dependence/psychosis |
| 1. Anemias | Iron deficiency or other/unspecified anemias and blood disease |
| 1. Arrhythmia | Specified arrhythmias and other heart rhythm disorders |
| 1. CHF | Congestive heart failure |
| 1. Cancer | Cancer |
| 1. Stroke | Stroke |
| 1. Brain_disorders | Dementia or other specified brain disorders |
| 1. Diabetes_w_comp | Diabetes Mellitus Complicated |
| 1. Endocrine_disorders | Other significant endocrine and metabolic disorders; disorders of fluid/electrolyte/acid base balance |
| 1. Liver_disease | Liver or biliary disease |
| 1. Malnutrition | Protein-calorie malnutrition |
| 1. Hemiplegia | Hemiplegia, paraplegia, paralysis, functional disability |
| 1. Peptic_ulcer | Peptic ulcer, hemorrhage, other specified gastrointestinal disorders |
| 1. Vascular | Vascular or circulatory disease |
| 1. Psychiatric_disorders | Major psychiatric disorders |
| 1. Hematological | Severe hematological disorders |
| 1. Valvular_disease | Valvular and Rheumatic Heart Disease |

**TKA/THA**

The initial set of comorbidities included 39 comorbidities generated from a union of the three comorbidity indices, plus 2 condition-specific comorbidities recommended by the clinicians, resulting in 41 comorbidities. The following feature-selection steps resulted in 11 comorbidities, that were used for the modeling:

1. Removed comorbidities with prevalence less than 1%, resulting in the following that were excluded, leaving 30 comorbidities:

| **Excluded Comorbidities** | **Label** |
| --- | --- |
| 1. Other_Hip_Cong_Def | Other congenital deformity of hip (joint) |
| 1. Post_Trau_Osteoarthritis | Post traumatic osteoarthritis |
| 1. Dialysis_status | Dialysis status (CC 134) |
| 1. Blood_Loss_Anemia | Blood Loss Anemia |
| 1. Alcohol_Abuse | Alcohol Abuse |
| 1. Drug_Abuse | Drug Abuse |
| 1. HIV_AIDS | HIV/AIDS |
| 1. Metastatic_cancer | Metastatic cancer or acute leukemia |
| 1. Hemiplegia | Hemiplegia, paraplegia, paralysis, functional disability |
| 1. Liver_disease | Liver disease |
| 1. Peptic_ulcer | Peptic Ulcer Disease |

1. Measured the OR of each comorbidity for readmission (at the .05 level corrected for multiple testing with Bonferroni), leaving all 30 comorbidities that had significant associations with readmission
2. Conducted a two-way co-occurrence test resulting in none being excluded.
3. Conducted a two-way directionality test, resulting in the following that were excluded leaving 19 variables that were involved in one or more significant direction tests:

| **Excluded Comorbidities** | **Label** |
| --- | --- |
| 1. Brain_disorders | Dementia or other specified brain disorders |
| 1. Cancer | Cancer |
| 1. Cellulitis | Cellulitis, local skin infection (CC 164) |
| 1. Deficiency_Anemia | Deficiency Anemia |
| 1. Diab_wo_comp | Diabetes mellitus |
| 1. Hematological | Severe hematological disorders |
| 1. Malnutrition | Protein-calorie malnutrition |
| 1. Neuro_disorders | Other Neurological Disorders |
| 1. Stroke | Stroke |
| 1. Ulcer | Decubitus ulcer or chronic skin ulcer (CC 157-161) |
| 1. Valvular_disease | Valvular Disease |

1. Repeated steps 2 through 4 in the replicate dataset resulting in 11 comorbidities shown below:

| **Final Comorbidities** | **Label** |
| --- | --- |
| 1. Arrhythmia | Specified arrhythmias and other heart rhythm disorders |
| 1. CHF | Congestive heart failure |
| 1. COPD | Chronic Obstructive Pulmonary Disease |
| 1. Coronary_angina | Coronary atherosclerosis or angina |
| 1. Endocrine_disorders | Other significant endocrine and metabolic disorders; disorders of fluid/electrolyte/acidbase balance |
| 1. Hypertension_Comp | Hypertension complicated |
| 1. Hypertension_Uncomp | Hypertension Uncomplicated |
| 1. Major_Symp_Abnormalities | Major symptoms, abnormalities (CC 178) |
| 1. Morbid_OB | Morbid obesity |
| 1. Psychiatric_disorders | Major psychiatric disorders |
| 1. Renal_failure | Renal failure (CC 135-140) |
