# Supplementary material for: A Framework for Modeling and Interpreting Patient Subgroups Applied to Hospital Readmission: Visual Analytical Approach
Source: JMIR Med Inform. 2022 Dec 7;10(12):e37239. doi: 10.2196/37239 (PMC9773032; doi:10.2196/37239)
Supplement: Multimedia Appendix 4 [file medinform_v10i12e37239_app4.docx]

## APPENDIX-4

## Classification Modeling

**Multinomial Logistic Regression Coefficients**

**COPD**

|  | **Bicluster-2** | **Bicluster-3** | **Bicluster-4** |
| --- | --- | --- | --- |
| (Intercept) | 9.478442033 | -15.60229477 | -20.58911845 |
| MV | 15.37003888 | 31.79526753 | 174.7625575 |
| Apnea | 14.73866236 | 30.13129952 | 174.8192981 |
| Infection | 13.07141525 | 30.94167533 | 175.7976241 |
| GI_other | -138.0330506 | -122.1606071 | -141.8251611 |
| Depression | 11.44920157 | 194.57748 | 15.50399067 |
| Psych_other | 6.633772193 | 193.1120262 | 15.01395622 |
| Neuropathy | 4.22511563 | 191.7033722 | 11.82453364 |
| CardioRespShock | 15.935738 | 30.78720915 | 177.4810561 |
| Coronary_angina | -140.3504037 | -121.6295958 | -139.1018879 |
| HD_other | 12.65500876 | 22.79910161 | 173.1189166 |
| Pneu | 12.40491598 | 192.8944261 | 14.40776138 |
| Ulcer | 25.40389722 | 36.65033091 | 174.4945337 |
| Cellulitis | 10.19338572 | 25.35264195 | 174.1779677 |
| Valvular_Disease | 179.470279 | 29.79933499 | 12.07687072 |
| Hypertension_Uncomp | -144.0822947 | -135.252917 | -149.1763872 |
| Hypertension_comp | 169.9103199 | 9.909139715 | -15.01176101 |
| Neurological_Disorders | 11.98793007 | 27.3962048 | 174.152075 |
| Diabetes_w_comp | 179.9458627 | 32.79407674 | 10.94569788 |
| Malnutrition | 2.619696434 | 190.2553747 | 10.57634814 |
| Morbid_OB | -144.7853066 | -126.4723011 | -150.75158 |
| Endocrine_disorder | 18.25087715 | 30.17692505 | 173.8734803 |
| Peptic_ulcer | 14.57401571 | 33.3138924 | 174.6959617 |
| Anemias | 180.37182 | 32.01649034 | 11.85268168 |
| Psychosis | 0.808153461 | 190.136092 | 8.470673123 |
| Hemiplegia | 22.05837142 | 36.91425527 | 173.7515174 |
| Heart_failure | 179.4801884 | 29.61286201 | 6.090102717 |
| Coronary_syndrome | 17.65746601 | 31.18056088 | 177.0738381 |
| Arrhythmias | -140.5676174 | -122.0347651 | -139.1832525 |
| Vascular | 12.82066036 | 29.47640378 | 174.8331118 |
| Renal_failure | 181.4635165 | 35.07327525 | 9.594086268 |

**CHF**

|  | **Bicluster-2** | **Bicluster-3** | **Bicluster-4** |
| --- | --- | --- | --- |
| (Intercept) | 4.228411649 | 3.221587055 | 4.404809652 |
| CABG | -16.88332455 | -16.46705046 | -16.90410559 |
| GI_other | 1.608383181 | 18.42437593 | 2.537152385 |
| Depression | 1.744237257 | 18.57833534 | 2.132954431 |
| Psych_other | 1.565344584 | 18.89075678 | 1.851558983 |
| CardioRespShock | 1.899086259 | 2.614460813 | 20.82096349 |
| Coronary_angina | -16.52943558 | -16.50069914 | -16.28824682 |
| HD_other | 1.630347764 | 1.900983651 | 20.69135601 |
| COPD | 2.208517874 | 18.8515632 | 2.812111665 |
| Pneu | 1.368125374 | 1.230710494 | 20.35722646 |
| Dialysis_status | 21.64550697 | 7.573268069 | 6.356824836 |
| Renal_failure | 16.64635654 | 3.207105993 | 2.875810167 |
| Nephritis | 24.05426922 | 11.91409993 | 8.430710694 |
| Other_uri_tract_disorders | 17.28359996 | 3.468138309 | 2.68316497 |
| Ulcer | 18.62035155 | 4.612512833 | 4.621717632 |
| Hypertension_Uncomp | -16.17798732 | -17.43341906 | -17.17899564 |
| Hypertension_Comp | 18.21349274 | 2.666169079 | 2.719974312 |
| Neuro_Disorders | 3.174148638 | 3.426243554 | 21.7581539 |
| Hypothyroidism | 1.532038616 | 18.6465425 | 2.742755582 |
| Obesity | 16.77179346 | 3.477665718 | 1.851913596 |
| Cancer | 1.825845108 | 18.78533294 | 2.280566193 |
| Diabetes_w_comp | 16.97604199 | 3.131645807 | 2.25066864 |
| Malnutrition | 1.654276729 | 2.26075743 | 20.16266194 |
| Endocrine_disorder | 16.95913583 | 3.414523477 | 2.762293049 |
| Liver_disease | 0.509273487 | 1.346254075 | 19.37619302 |
| Peptic_ulcer | 1.882489155 | 2.868064904 | 20.99453939 |
| Hematological | 1.215479083 | 1.623296079 | 20.18186306 |
| Anemias | 16.84448889 | 2.650113464 | 2.132489404 |
| Brain_disorders | 1.214570676 | 19.0044895 | 2.119832832 |
| Psychosis | 1.612149611 | 18.59666356 | 2.038965354 |
| Psychiatric_disorders | 3.533134983 | 19.8653935 | 3.164125739 |
| Hemiplegia | 2.634196819 | 3.196095963 | 21.40555332 |
| CHF | -16.83591928 | -17.14949663 | -16.56386079 |
| Coronary_syndrome | 1.423033472 | 2.813198347 | 20.7510375 |
| Valvular_disease | -16.84402957 | -16.55559304 | -16.67291059 |
| Arrhythmia | -17.41765184 | -17.22878444 | -17.44203684 |
| Stroke | 1.248759632 | 1.486455436 | 19.78749343 |
| Vascular | 0.908845182 | 1.356092137 | 19.93213936 |

**TKA/THA**

|  | **Bicluster-2** | **Bicluster-3** | **Bicluster-4** | **Bicluster-5** | **Bicluster-6** | **Bicluster-7** |
| --- | --- | --- | --- | --- | --- | --- |
| (Intercept) | -645.18 | 19.65 | -365.25 | -1.65 | 154.42 | 162.00 |
| Renal_failure | 568.98 | 529.82 | 1385.51 | 811.46 | 748.18 | 514.28 |
| Major_Symp_Abnormalities | 338.07 | 756.45 | 494.57 | 542.41 | 548.90 | 529.61 |
| Hypertension_Uncomp | -863.03 | -675.02 | -695.36 | -655.72 | -711.68 | -668.86 |
| Hypertension_Comp | -380.60 | -814.06 | 468.07 | -52.64 | -121.56 | -260.13 |
| Morbid_OB | 229.35 | 294.47 | 409.00 | 492.63 | 824.96 | 492.19 |
| Endocrine_disorders | 426.79 | 310.04 | 1280.41 | 702.34 | 612.09 | 439.19 |
| Psychiatric_disorders | -12.78 | 22.00 | 196.72 | -634.96 | -258.19 | 944.17 |
| CHF | 881.66 | 179.53 | 67.03 | -113.47 | -132.62 | 46.56 |
| Coronary_angina | 865.33 | 94.72 | 1.15 | -184.54 | -119.69 | -108.41 |
| Arrhythmia | 936.49 | 95.61 | 77.56 | -126.56 | -133.83 | -163.73 |
| COPD | 360.05 | 240.86 | 508.18 | 990.19 | 358.05 | 655.49 |

**Subgroup Risk**

**COPD.** The COPD dataset included 186041 total patients, of which 29026 were cases (15.6%). The following are the percentage of cases in each bicluster (subgroup risk) after classification of the 100% cases and 100% controls by the classification model, and then juxtaposed with the visualization of the respective patient subgroups:

| **Bicluster** | **Total Patients** | **Cases** | **Percent** | **CI95 (Min)** | **CI95 (Max)** |
| --- | --- | --- | --- | --- | --- |
| 1 | 76296 | 9673 | 12.7% | 12.4 | 12.9 |
| 2 | 43477 | 7731 | 17.8% | 17.4 | 18.1 |
| 3 | 37174 | 5906 | 15.9% | 15.5 | 16.3 |
| 4 | 29094 | 5716 | 19.6% | 19.2 | 20.1 |

**CHF.** The CHF dataset included 295761 total patients, of which 51573 were cases (17.4%). The following are the percentage of cases in each bicluster (subgroup risk) after classification of the 100% cases and 100% controls into subgroups by the classification model, and juxtaposed with the visualization of the respective patient subgroups:

| **Bicluster** | **Total Patients** | **Cases** | **Percent** | **CI95 (Min)** | **CI95 (Max)** |
| --- | --- | --- | --- | --- | --- |
| 1 | 9673 | 76296 | 12.7% | 12.4 | 12.9 |
| 2 | 7731 | 43477 | 17.8% | 17.4 | 18.1 |
| 3 | 5906 | 37174 | 15.9% | 15.5 | 16.3 |
| 4 | 5716 | 29094 | 19.6% | 19.2 | 20.1 |

**THA/TKA.** The TKA/THA dataset included 356772 total patients, of which 16520 were cases (4.6%). The following are the percentage of cases in each bicluster (subgroup risk) after classification of 100% cases and 100% controls by the classification model, and then juxtaposed with the visualization of the respective patient subgroups:

| **Bicluster** | **Total Patients** | **Cases** | **Percent** | **CI95 (Min)** | **CI95 (Max)** |
| --- | --- | --- | --- | --- | --- |
| 1 | 2157 | 57045 | 3.8% | 3.6 | 3.9 |
| 2 | 2317 | 33535 | 6.9% | 6.6 | 7.2 |
| 3 | 2255 | 55260 | 4.1% | 3.9 | 4.2 |
| 4 | 2135 | 29475 | 7.2% | 7 | 7.5 |
| 5 | 2003 | 30856 | 6.5% | 6.2 | 6.8 |
| 6 | 1945 | 43185 | 4.5% | 4.3 | 4.7 |
| 7 | 3708 | 107416 | 3.5% | 3.3 | 3.6 |
