# Supplementary material for: A Framework for Modeling and Interpreting Patient Subgroups Applied to Hospital Readmission: Visual Analytical Approach
Source: JMIR Med Inform. 2022 Dec 7;10(12):e37239. doi: 10.2196/37239 (PMC9773032; doi:10.2196/37239)
Supplement: Multimedia Appendix 5 [file medinform_v10i12e37239_app5.docx]

## APPENDIX-5

## Predictive Modeling

**COPD**

**Discrimination.** The following are box plots of risk prediction by readmission status for the Standard Model and the Hierarchical Model.


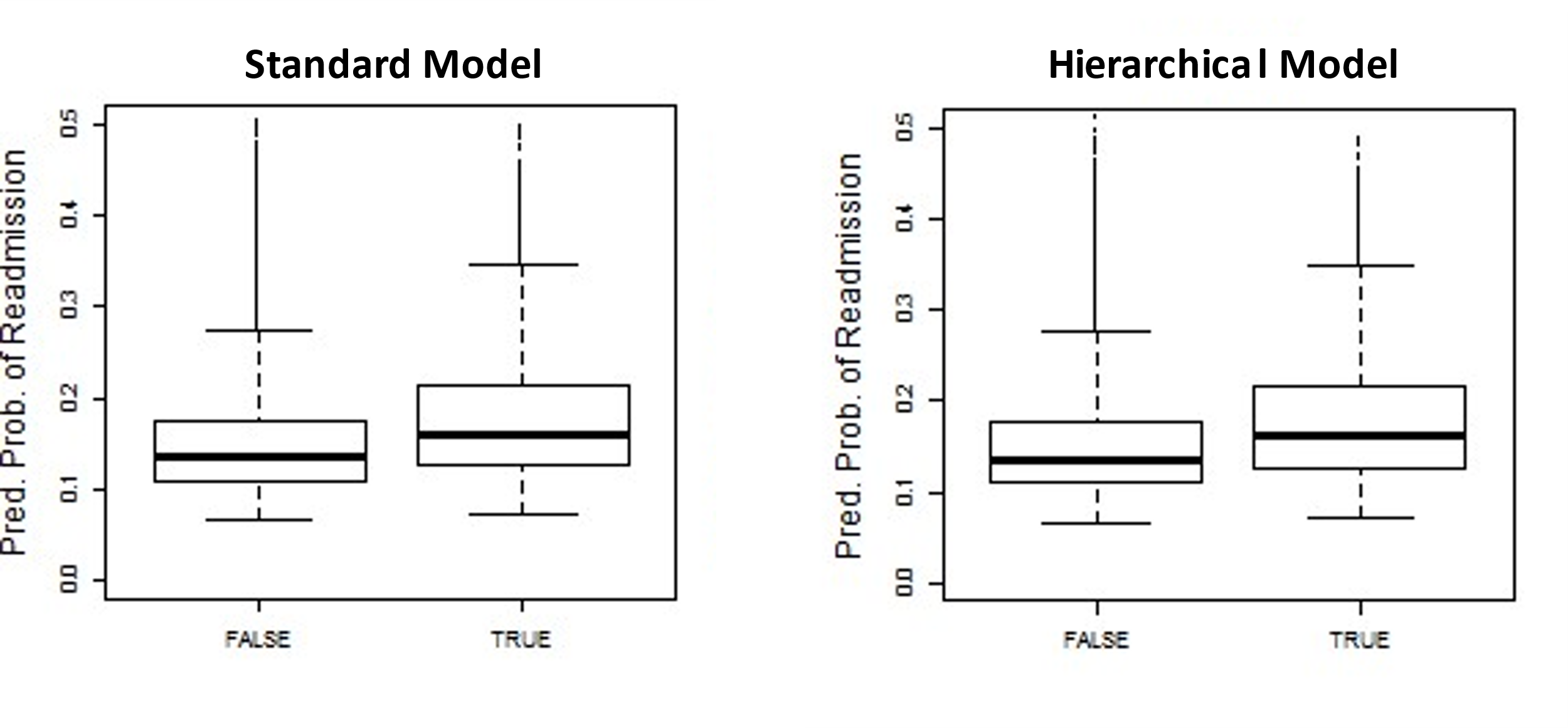


**Calibration.** The following are calibration plots showing logistic regressions relating readmission status to the logistic quantile of the predicted probability of readmission yield regression lines with specified intercept and slope (the ideal regression line would have intercept=0 and slope=1, which is shown shaded for reference). The histograms at the bottom of each graph reflect the frequency of modeled data, horizontal axes show predicted probability, vertical axes show actual probability, and the axes of all figures are constrained to range from 0 to .5.


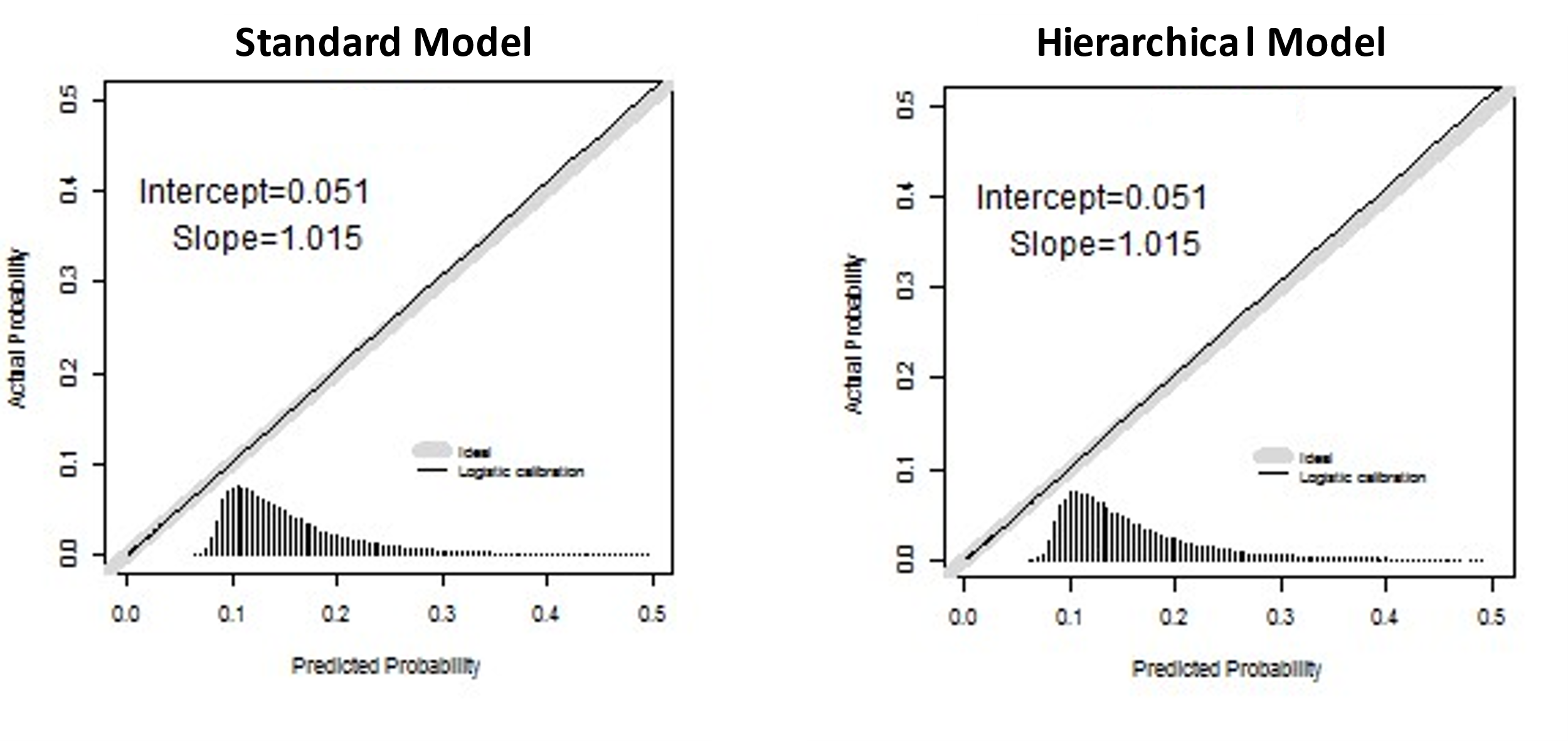


**Coefficients.** Logistic regression coefficients relating readmission status to the logistic quantile of the predicted probability of readmission for each model, with standard errors.

|  | **Intercept (Standard Error)** | **Slope (Standard Error)** |
| --- | --- | --- |
| **Standard Model** | 0.051 (0.049) | 1.015 (0.029) |
| **Hierarchical Model** | 0.051 (0.049) | 1.015 (0.029) |

**Model Coefficients for Standard Model (COPD)**

| (Intercept) | -2.136379215 |
| --- | --- |
| sex2 | -0.018974047 |
| AgeAdm | -0.006994626 |
| RACE51 | 0.295066886 |
| RACE52 | 0.341204445 |
| RACE53 | 0.171206943 |
| RACE55 | 0.273604446 |
| MV | 0.117480441 |
| Apnea | -0.012262741 |
| Infection | 0.026450984 |
| GI_other | 0.087222647 |
| Depression | 0.080231389 |
| Psych_other | 0.082623979 |
| Neuropathy | 0.069716803 |
| CardioRespShock | 0.228794257 |
| Coronary_angina | 0.048182744 |
| HD_other | 0.015511356 |
| Pneu | 0.057607182 |
| Ulcer | 0.091465998 |
| Cellulitis | 0.095049297 |
| Valvular_Disease | 0.078134984 |
| Hypertension_Uncomp | 0.087684427 |
| Hypertension_comp | 0.143660314 |
| Neurological_Disorders | 0.075023091 |
| Diabetes_w_comp | 0.077946469 |
| Malnutrition | 0.095555424 |
| Morbid_OB | -0.056028398 |
| Endocrine_disorder | 0.137464197 |
| Peptic_ulcer | 0.114498859 |
| Anemias | 0.1663521 |
| Psychosis | 0.13049134 |
| Hemiplegia | 0.113401107 |
| Heart_failure | 0.23205475 |
| Coronary_syndrome | 0.110911596 |
| Arrhythmias | 0.188301605 |
| Vascular | 0.090867219 |
| Renal_failure | 0.11154648 |

**Model Coefficients for Hierarchical Model (COPD)**

| (Intercept) | -2.170381336 |
| --- | --- |
| sex2 | -0.018743606 |
| AgeAdm | -0.00702047 |
| RACE51 | 0.29629354 |
| RACE52 | 0.342336002 |
| RACE53 | 0.172316947 |
| RACE55 | 0.275083731 |
| MV | 0.112639359 |
| Apnea | -0.017323465 |
| Infection | 0.02133693 |
| GI_other | 0.095383617 |
| Depression | 0.072418589 |
| Psych_other | 0.075096553 |
| Neuropathy | 0.063679331 |
| CardioRespShock | 0.224665848 |
| Coronary_angina | 0.056139938 |
| HD_other | 0.011352801 |
| Pneu | 0.052053617 |
| Ulcer | 0.089352719 |
| Cellulitis | 0.091640641 |
| Valvular_Disease | 0.074608627 |
| Hypertension_Uncomp | 0.100372891 |
| Hypertension_comp | 0.143513998 |
| Neurological_Disorders | 0.070642457 |
| Diabetes_w_comp | 0.076044197 |
| Malnutrition | 0.089847317 |
| Morbid_OB | -0.045581819 |
| Endocrine_disorder | 0.132138897 |
| Peptic_ulcer | 0.1109747 |
| Anemias | 0.162850779 |
| Psychosis | 0.124238457 |
| Hemiplegia | 0.109511728 |
| Heart_failure | 0.228425309 |
| Coronary_syndrome | 0.106258 |
| Arrhythmias | 0.19629312 |
| Vascular | 0.085350053 |
| Renal_failure | 0.106363156 |
| PredCluster2 | 0.042362651 |
| PredCluster3 | 0.05007554 |
| PredCluster4 | 0.052171757 |

**Standard Model and Hierarchical Model for COPD**

The following table shows C-statistics for the Standard Model and the Hierarchical Model.

|  | **C-Statistic** | **CI95 (Min)** | **CI95 (Max)** |
| --- | --- | --- | --- |
| **Standard Model** | 0.624 | 0.617 | 0.631 |
| **Hierarchical Model** | 0.625 | 0.618 | 0.632 |

The following table shows C-statistics for the Standard Model used to predict readmission for patients in each bicluster separately.

|  | **C-Statistic** | **CI95 (Min)** | **CI95 (Max)** |
| --- | --- | --- | --- |
| **Bicluster 1** | 0.596 | 0.584 | 0.608 |
| **Bicluster 2** | 0.610 | 0.596 | 0.624 |
| **Bicluster 3** | 0.616 | 0.600 | 0.632 |
| **Bicluster 4** | 0.636 | 0.620 | 0.652 |

**CMS Models (CMS Standard Model and CMS Hierarchical Model) for COPD**

The following table shows C-statistics for the CMS Standard Model and the CMS Hierarchical Model.

|  | **C-Statistic** | **CI95 (Min)** | **CI95 (Max)** |
| --- | --- | --- | --- |
| **CMS Standard Model** | 0.622 | 0.615 | 0.629 |
| **CMS Hierarchical Model** | 0.622 | 0.615 | 0.629 |

The following table shows C-statistics for the CMS Standard Model used to predict readmission for patients in each bicluster separately.

|  | **C-Statistic** | **CI95 (Min)** | **CI95 (Max)** |
| --- | --- | --- | --- |
| **Bicluster 1** | 0.587 | 0.575 | 0.599 |
| **Bicluster 2** | 0.611 | 0.598 | 0.625 |
| **Bicluster 3** | 0.620 | 0.604 | 0.636 |
| **Bicluster 4** | 0.638 | 0.622 | 0.654 |

**Model Coefficients for CMS Standard Model (COPD)** [42]

| (Intercept) | -2.188594326 |
| --- | --- |
| V1 | -0.004884898 |
| V2 | 0.173933436 |
| V3 | -0.004371274 |
| V4 | 0.014983286 |
| V5 | 0.158622714 |
| V6 | 0.197266795 |
| V7 | 0.082435098 |
| V8 | -0.042473792 |
| V9 | 0.075588823 |
| V10 | 0.089748913 |
| V11 | -0.042615016 |
| V12 | 0.146684496 |
| V13 | 0.381543598 |
| V14 | 0.085139806 |
| V15 | 0.075183842 |
| V16 | 0.114503012 |
| V17 | 0.16450527 |
| V18 | -0.034979967 |
| V19 | 0.198698509 |
| V20 | 0.100948646 |
| V21 | 0.069808662 |
| V22 | 0.023636931 |
| V23 | 0.069163157 |
| V24 | 0.111452974 |
| V25 | 0.064113847 |
| V26 | -0.039814801 |
| V27 | 0.173239512 |
| V28 | 0.212336424 |
| V29 | 0.066160516 |
| V30 | 0.109441845 |
| V31 | 0.190317991 |
| V32 | 0.043561901 |
| V33 | 0.015612425 |
| V34 | 0.074089074 |
| V35 | 0.069126003 |
| V36 | 0.036551409 |
| V37 | 0.174960292 |
| V38 | 0.102815442 |
| V39 | 0.082962429 |
| V40 | 0.127491216 |

**Model Coefficients for CMS Hierarchical Model (COPD)**

| (Intercept) | -2.222560918 |
| --- | --- |
| V1 | -0.005037176 |
| V2 | 0.164652122 |
| V3 | -0.011571161 |
| V4 | 0.007278066 |
| V5 | 0.159430966 |
| V6 | 0.197452639 |
| V7 | 0.083353046 |
| V8 | -0.042706545 |
| V9 | 0.071953525 |
| V10 | 0.098535986 |
| V11 | -0.030172663 |
| V12 | 0.143262108 |
| V13 | 0.382813245 |
| V14 | 0.081387202 |
| V15 | 0.086119119 |
| V16 | 0.11252111 |
| V17 | 0.150832777 |
| V18 | -0.035543286 |
| V19 | 0.207400734 |
| V20 | 0.10019542 |
| V21 | 0.07625943 |
| V22 | 0.023819484 |
| V23 | 0.076936963 |
| V24 | 0.105746192 |
| V25 | 0.07104448 |
| V26 | -0.033998175 |
| V27 | 0.168407005 |
| V28 | 0.202506355 |
| V29 | 0.063304577 |
| V30 | 0.116819091 |
| V31 | 0.195860925 |
| V32 | 0.037067697 |
| V33 | 0.014270778 |
| V34 | 0.066636971 |
| V35 | 0.069127715 |
| V36 | 0.043952171 |
| V37 | 0.129843017 |
| V38 | 0.099065392 |
| V39 | 0.078360538 |
| V40 | 0.127042521 |
| PredCluster2 | 0.098236779 |
| PredCluster3 | 0.014085719 |
| PredCluster4 | 0.072649738 |

**CHF**

**Discrimination.** The following are box plots of risk prediction by readmission status for the Standard Model and the Hierarchical Model.


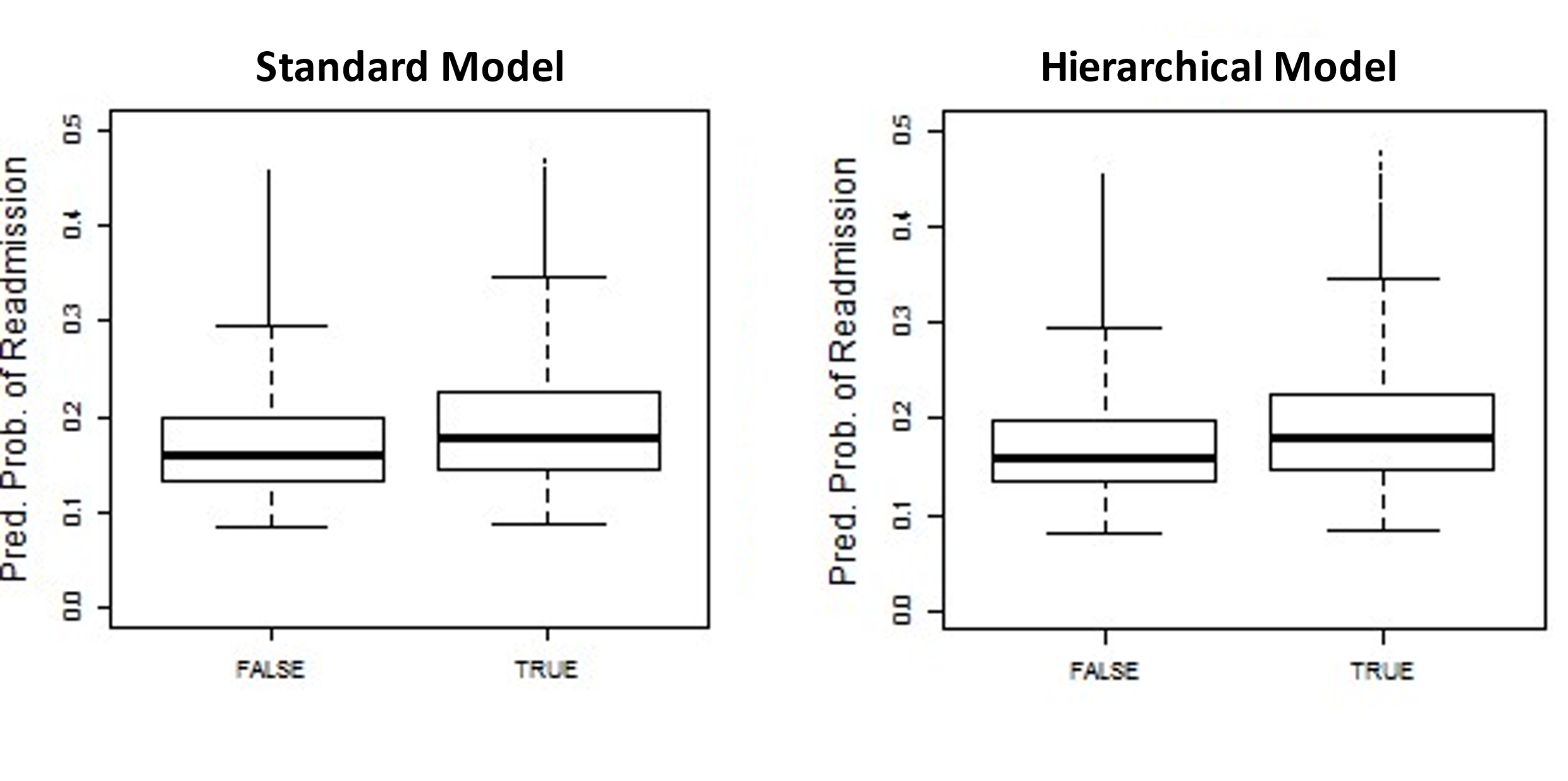


**Calibration.** The following are calibration plots showing logistic regressions relating readmission status to the logistic quantile of the predicted probability of readmission yield regression lines with specified intercept and slope (the ideal regression line would have intercept=0 and slope=1, which is shown shaded for reference). The histograms at the bottom of each graph reflect the frequency of modeled data, horizontal axes show predicted probability, vertical axes show actual probability, and the axes of all figures are constrained to range from 0 to .5.


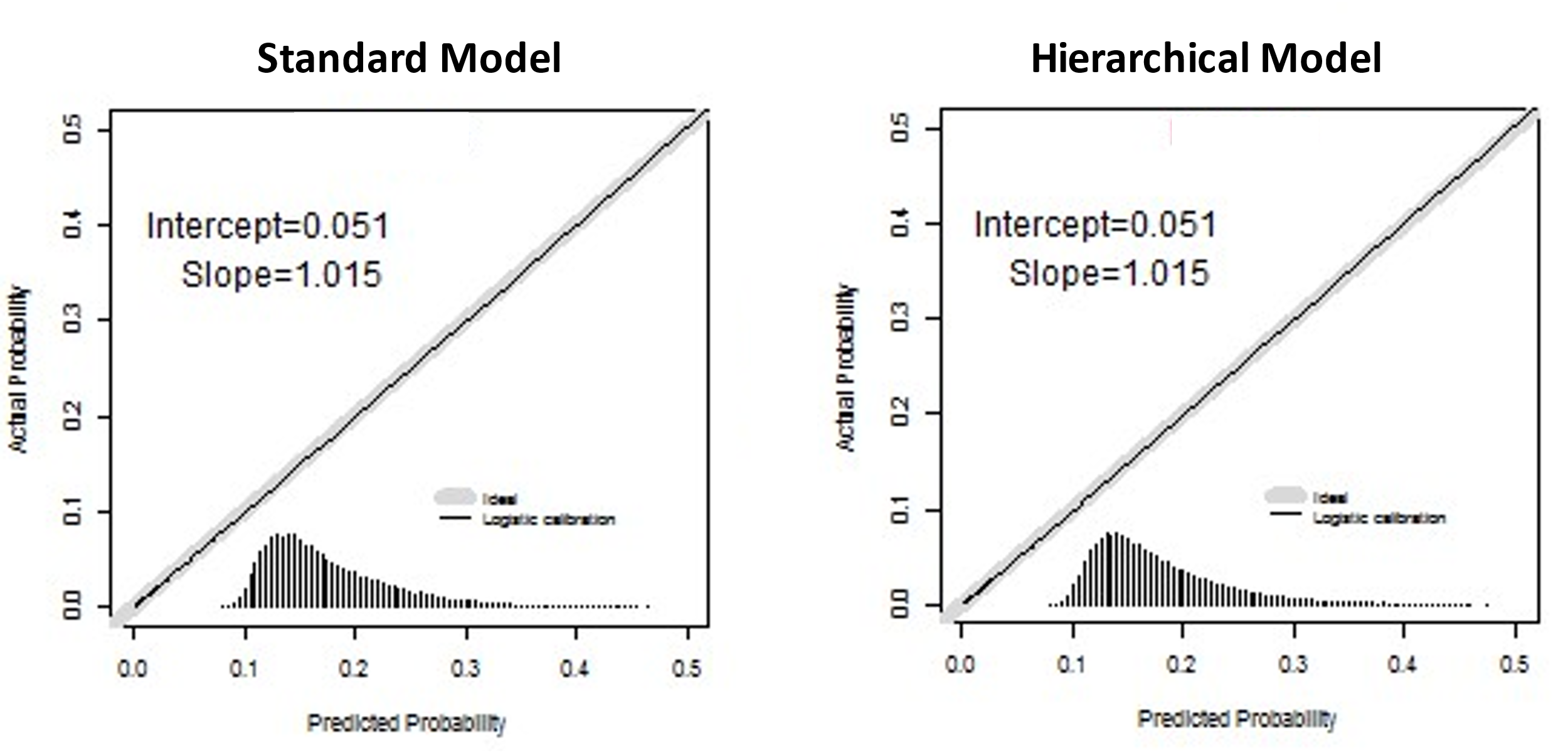


**Coefficients.** The following are the logistic regression coefficients relating readmission status to the logistic quantile of the predicted probability of readmission for each model, with standard errors.

|  | **Intercept (Standard Error)** | **Slope (Standard Error)** |
| --- | --- | --- |
| **Standard Model** | .009 (0.042) | 1.013 (0.027) |
| **Hierarchical Model** | 0.008 (0.042) | 1.013 (0.027) |

**Model Coefficients for Standard Model (CHF)**

| (Intercept) | -1.475389967 |
| --- | --- |
| sex2 | 0.074653204 |
| AgeAdm | -0.008514725 |
| RACE51 | -0.036053802 |
| RACE52 | 0.040134152 |
| RACE53 | -0.079761067 |
| RACE55 | 0.072722149 |
| CABG | 0.030894001 |
| GI_other | 0.056464897 |
| Depression | 0.025005144 |
| Psych_other | 0.045337378 |
| CardioRespShock | 0.048277891 |
| Coronary_angina | 0.055087252 |
| HD_other | 0.080761149 |
| COPD | 0.149282237 |
| Pneu | 0.081351108 |
| Dialysis_status | 0.173258929 |
| Renal_failure | 0.140937411 |
| Nephritis | 0.012307007 |
| Other_uri_tract_disorders | 0.065712737 |
| Ulcer | 0.108579462 |
| Hypertension_Uncomp | 0.041031817 |
| Hypertension_Comp | 0.105063607 |
| Neuro_Disorders | 0.032623414 |
| Hypothyroidism | 0.024951326 |
| Obesity | 0.026406255 |
| Cancer | 0.028160953 |
| Diabetes_w_comp | 0.085693489 |
| Malnutrition | 0.038386944 |
| Endocrine_disorder | 0.093422704 |
| Liver_disease | 0.051990801 |
| Peptic_ulcer | 0.053199182 |
| Hematological | 0.045559899 |
| Anemias | 0.123037859 |
| Brain_disorders | -0.00749815 |
| Psychosis | 0.093703137 |
| Psychiatric_disorders | 0.072868486 |
| Hemiplegia | 0.057172104 |
| CHF | 0.106985057 |
| Coronary_syndrome | 0.096953224 |
| Valvular_disease | 0.018311001 |
| Arrhytmia | 0.00786506 |
| Stroke | -0.012731604 |
| Vascular | 0.061193164 |

**Model Coefficients for Hierarchical Model (CHF)**

| (Intercept) | -1.449848979 |
| --- | --- |
| sex2 | 0.074706479 |
| AgeAdm | -0.008504158 |
| RACE51 | -0.035610416 |
| RACE52 | 0.04010896 |
| RACE53 | -0.079311376 |
| RACE55 | 0.073284464 |
| CABG | 0.024674808 |
| GI_other | 0.061045731 |
| Depression | 0.029184869 |
| Psych_other | 0.049224598 |
| CardioRespShock | 0.057426429 |
| Coronary_angina | 0.048052865 |
| HD_other | 0.089668573 |
| COPD | 0.154094768 |
| Pneu | 0.089524374 |
| Dialysis_status | 0.164539306 |
| Renal_failure | 0.139877836 |
| Nephritis | 0.004910838 |
| Other_uri_tract_disorders | 0.062840363 |
| Ulcer | 0.105143831 |
| Hypertension_Uncomp | 0.032829812 |
| Hypertension_Comp | 0.100497599 |
| Neuro_Disorders | 0.040022189 |
| Hypothyroidism | 0.029106384 |
| Obesity | 0.022538905 |
| Cancer | 0.031985591 |
| Diabetes_w_comp | 0.082993693 |
| Malnutrition | 0.046167614 |
| Endocrine_disorder | 0.091753086 |
| Liver_disease | 0.060002993 |
| Peptic_ulcer | 0.061215338 |
| Hematological | 0.054764671 |
| Anemias | 0.12180882 |
| Brain_disorders | -0.003163932 |
| Psychosis | 0.097984879 |
| Psychiatric_disorders | 0.075834723 |
| Hemiplegia | 0.065565413 |
| CHF | 0.10067029 |
| Coronary_syndrome | 0.107487626 |
| Valvular_disease | 0.012129124 |
| Arrhytmia | 0.001138884 |
| Stroke | -0.003992176 |
| Vascular | 0.069322294 |
| PredCluster2 | -0.013499297 |
| PredCluster3 | -0.039541727 |
| PredCluster4 | -0.057008659 |

**Standard Model and Hierarchical Model for CHF**

The following table shows C-statistics for the Standard Model and the Hierarchical Model.

|  | **C-Statistic** | **CI95 (Min)** | **CI95 (Max)** |
| --- | --- | --- | --- |
| **Standard Model** | 0.600 | 0.595 | 0.605 |
| **Hierarchical Model** | 0.600 | 0.595 | 0.606 |

The following table shows C-statistics for the Standard Model used to predict readmission for patients in each bicluster separately.

|  | **C-Statistic** | **CI95 (Min)** | **CI95 (Max)** |
| --- | --- | --- | --- |
| **Bicluster 1** | 0.570 | 0.560 | 0.580 |
| **Bicluster 2** | 0.587 | 0.576 | 0.598 |
| **Bicluster 3** | 0.597 | 0.585 | 0.608 |
| **Bicluster 4** | 0.614 | 0.602 | 0.625 |

**CMS Models (CMS Standard Model and CMS Hierarchical Model) for CHF**

The following table shows C-statistics for the CMS Standard Model and the CMS Hierarchical Model.

|  | **C-Statistic** | **CI95 (Min)** | **CI95 (Max)** |
| --- | --- | --- | --- |
| **CMS Standard Model** | 0.602 | 0.597 | 0.608 |
| **CMS Hierarchical Model** | 0.602 | 0.597 | 0.608 |

The following table shows C-statistics for the CMS Standard Model used to predict readmission for patients in each bicluster separately.

|  | **C-Statistic** | **CI95 (Min)** | **CI95 (Max)** |
| --- | --- | --- | --- |
| **Bicluster 1** | 0.573 | 0.563 | 0.583 |
| **Bicluster 2** | 0.590 | 0.579 | 0.600 |
| **Bicluster 3** | 0.603 | 0.591 | 0.614 |
| **Bicluster 4** | 0.614 | 0.603 | 0.626 |

**Model Coefficients for CMS Standard Model (CHF)** [43]

| (Intercept) | -2.062052423 |
| --- | --- |
| V1 | -0.008807384 |
| V2 | 0.08069205 |
| V3 | 0.026511089 |
| V4 | 0.059292935 |
| V5 | 0.021486724 |
| V6 | 0.083551993 |
| V7 | 0.043335937 |
| V8 | 0.110433094 |
| V9 | 0.050644153 |
| V10 | 0.055051891 |
| V11 | 0.063148831 |
| V12 | 0.046434593 |
| V13 | 0.130099365 |
| V14 | -0.001401658 |
| V15 | 0.096204102 |
| V16 | 0.074628886 |
| V17 | 0.02554376 |
| V18 | 0.045885408 |
| V19 | 0.072014832 |
| V20 | 0.053421316 |
| V21 | 0.124207717 |
| V22 | 0.105487347 |
| V23 | 0.051450424 |
| V24 | 0.021372813 |
| V25 | 0.01075549 |
| V26 | 0.086498498 |
| V27 | -0.001335018 |
| V28 | 0.070577466 |
| V29 | 0.142129454 |
| V30 | 0.046599464 |
| V31 | 0.042236327 |
| V32 | 0.07795193 |
| V33 | 0.206227519 |
| V34 | 0.170726009 |
| V35 | 0.05424802 |
| V36 | 0.071097061 |
| V37 | 0.117969103 |

**Model Coefficients for CMS Hierarchical Model (CHF)**

| (Intercept) | -2.050961429 |
| --- | --- |
| V1 | -0.008696382 |
| V2 | 0.080417206 |
| V3 | 0.024568048 |
| V4 | 0.060401182 |
| V5 | 0.026470131 |
| V6 | 0.07824069 |
| V7 | 0.055357171 |
| V8 | 0.100353589 |
| V9 | 0.061970384 |
| V10 | 0.066594667 |
| V11 | 0.068254076 |
| V12 | 0.058624423 |
| V13 | 0.120852698 |
| V14 | 0.005596376 |
| V15 | 0.102047312 |
| V16 | 0.079661612 |
| V17 | 0.031011406 |
| V18 | 0.050815364 |
| V19 | 0.084441452 |
| V20 | 0.065024208 |
| V21 | 0.119511648 |
| V22 | 0.11654221 |
| V23 | 0.047832743 |
| V24 | 0.0189412 |
| V25 | 0.007449149 |
| V26 | 0.097154811 |
| V27 | 0.009679744 |
| V28 | 0.079037213 |
| V29 | 0.147713008 |
| V30 | 0.04662239 |
| V31 | 0.041108317 |
| V32 | 0.088384596 |
| V33 | 0.190553921 |
| V34 | 0.160246633 |
| V35 | 0.035498901 |
| V36 | 0.061096207 |
| V37 | 0.106604226 |
| PredCluster2 | 0.028721208 |
| PredCluster3 | -0.029432347 |
| PredCluster4 | -0.048839832 |

**TKA/THA**

**Discrimination.** The following are box plots of risk prediction by readmission status for the Standard Model and the Hierarchical Model.


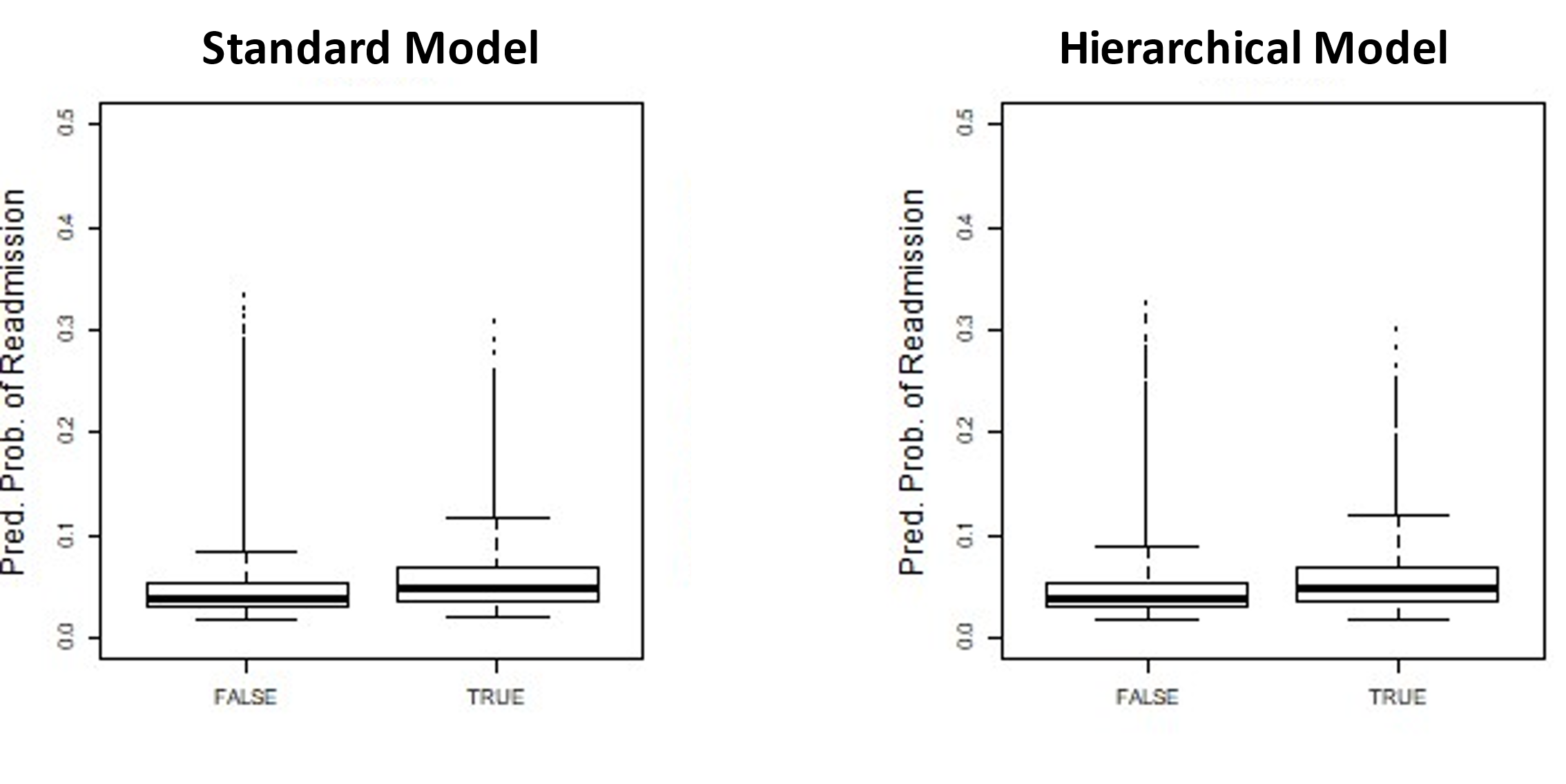


**Calibration.** The following are calibration plots showing logistic regressions relating readmission status to the logistic quantile of the predicted probability of readmission yield regression lines with specified intercept and slope (the ideal regression line would have intercept=0 and slope=1, which is shown shaded for reference). The histograms at the bottom of each graph reflect the frequency of modeled data, horizontal axes show predicted probability, vertical axes show actual probability, and the axes of all figures are constrained to range from 0 to .5.


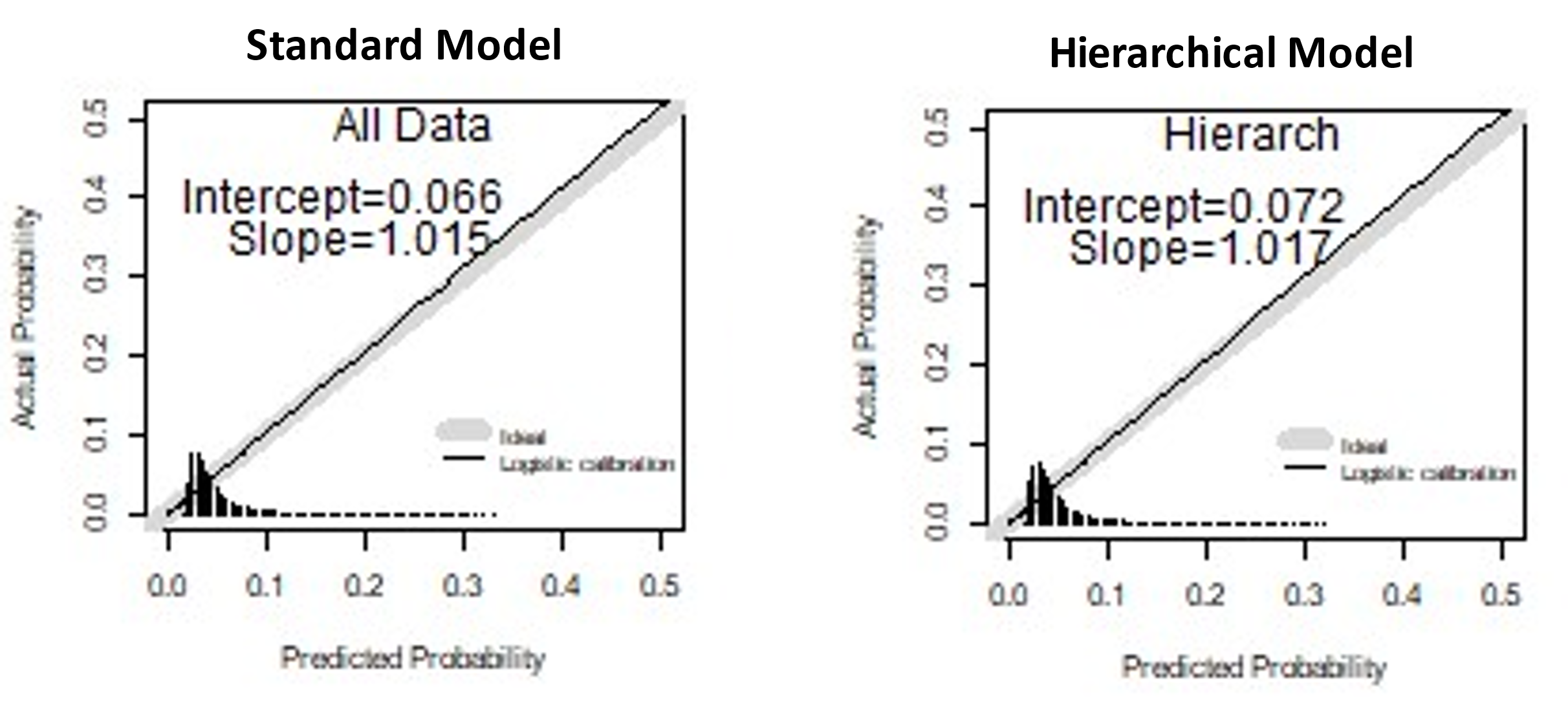


**Coefficients.** The following are logistic regression coefficients relating readmission status to the logistic quantile of the predicted probability of readmission for each model, with standard errors.

|  | **Intercept (Standard Error)** | **Slope (Standard Error)** |
| --- | --- | --- |
| **Standard Model** | 0.066 (0.092) | 1.015 (0.031) |
| **Hierarchical Model** | 0.072 (0.092) | 1.017 (0.031) |

**Model Coefficients for Standard Model (TKA/THA)**

| (Intercept) | -6.263520554 |
| --- | --- |
| sex2 | -0.156977542 |
| AgeAdm | 0.037561114 |
| RACE51 | 0.107946335 |
| RACE52 | 0.385560747 |
| RACE53 | 0.083620721 |
| RACE55 | 0.103221123 |
| Renal_failure | 0.367756519 |
| Major_Symp_Abnormalities | 0.173001919 |
| Hypertension_Uncomp | 0.115321908 |
| Hypertension_Comp | 0.034487102 |
| Morbid_OB | 0.1284828 |
| Endocrine_disorders | 0.221828594 |
| Psychiatric_disorders | 0.321401406 |
| CHF | 0.21488135 |
| Coronary_angina | 0.263264479 |
| Arrhytmia | 0.24838679 |
| COPD | 0.389660791 |

**Model Coefficients for Hierarchical Model (TKA/THA)**

| (Intercept) | -6.257678633 |
| --- | --- |
| sex2 | -0.156625896 |
| AgeAdm | 0.037256943 |
| RACE51 | 0.104795822 |
| RACE52 | 0.38085003 |
| RACE53 | 0.080648574 |
| RACE55 | 0.100025632 |
| Renal_failure | 0.326415209 |
| Major_Symp_Abnormalities | 0.143870143 |
| Hypertension_Uncomp | 0.109878666 |
| Hypertension_Comp | 0.028345431 |
| Morbid_OB | 0.068228389 |
| Endocrine_disorders | 0.209295683 |
| Psychiatric_disorders | 0.439980871 |
| CHF | 0.241863504 |
| Coronary_angina | 0.242997744 |
| Arrhytmia | 0.236970594 |
| COPD | 0.294032113 |
| PredCluster2 | 0.101832731 |
| PredCluster3 | 0.060642002 |
| PredCluster4 | 0.117407922 |
| PredCluster5 | 0.201783265 |
| PredCluster6 | 0.14290807 |
| PredCluster7 | -0.0369165 |

**Standard Model and Hierarchical Model for THA/TKA**

The following table shows C-statistics for the Standard Model and the Hierarchical Model.

|  | **C-Statistic** | **CI95 (Min)** | **CI95 (Max)** |
| --- | --- | --- | --- |
| **Standard Model** | 0.638 | 0.629 | 0.646 |
| **Hierarchical Model** | 0.638 | 0.629 | 0.647 |

The following table shows C-statistics for the Standard Model used to predict readmission for patients in each bicluster separately.

|  | **C-Statistic** | **CI95 (Min)** | **CI95 (Max)** |
| --- | --- | --- | --- |
| **Bicluster 1** | 0.578 | 0.553 | 0.603 |
| **Bicluster 2** | 0.608 | 0.584 | 0.631 |
| **Bicluster 3** | 0.595 | 0.570 | 0.620 |
| **Bicluster 4** | 0.610 | 0.585 | 0.635 |
| **Bicluster 5** | 0.584 | 0.559 | 0.609 |
| **Bicluster 6** | 0.579 | 0.553 | 0.606 |
| **Bicluster 7** | 0.636 | 0.617 | 0.655 |

**CMS Models (CMS Standard Model and CMS Hierarchical Model) for TKA/THA**

**C-Statistics.** The following table shows C-statistics for the CMS Standard Model and the CMS Hierarchical Model.

|  | **C-Statistic** | **CI95 (Min)** | **CI95 (Max)** |
| --- | --- | --- | --- |
| **CMS Standard Model** | 0.648 | 0.640 | 0.657 |
| **CMS Hierarchical Model** | 0.648 | 0.640 | 0.657 |

The following table shows C-statistics for the CMS Standard Model used to predict readmission for patients in each bicluster separately.

|  | **C-Statistic** | **CI95 (Min)** | **CI95 (Max)** |
| --- | --- | --- | --- |
| **Bicluster 1** | 0.585 | 0.561 | 0.609 |
| **Bicluster 2** | 0.632 | 0.609 | 0.655 |
| **Bicluster 3** | 0.617 | 0.594 | 0.641 |
| **Bicluster 4** | 0.630 | 0.606 | 0.653 |
| **Bicluster 5** | 0.608 | 0.583 | 0.632 |
| **Bicluster 6** | 0.589 | 0.562 | 0.615 |
| **Bicluster 7** | 0.640 | 0.621 | 0.659 |

**Model Coefficients for CMS Standard Model (TKA/THA)** [44]

| (Intercept) | -4.007625537 |
| --- | --- |
| Var1 | 0.036039292 |
| Var2 | -0.149437339 |
| Var3 | 0.088157516 |
| Var4 | 0.252326647 |
| Var5 | -0.255229777 |
| Var6 | 0.234030925 |
| Var7 | 0.131248955 |
| Var8 | 0.118415513 |
| Var9 | -0.014234681 |
| Var10 | 0.158442117 |
| Var11 | 0.186149829 |
| Var12 | 0.10822119 |
| Var13 | 0.120553114 |
| Var14 | 0.213179137 |
| Var15 | 0.166404922 |
| Var16 | 0.244882944 |
| Var17 | 0.260963406 |
| Var18 | 0.228775313 |
| Var19 | 0.099773803 |
| Var20 | 0.167375189 |
| Var21 | 0.236389437 |
| Var22 | 0.08572084 |
| Var23 | 0.22805813 |
| Var24 | 0.092669018 |
| Var25 | 0.099767352 |
| Var26 | 0.363700358 |
| Var27 | 0.038962561 |
| Var28 | 0.582409201 |
| Var29 | 0.317306501 |
| Var30 | 0.103490531 |
| Var31 | -0.001329201 |
| Var32 | 0.026795269 |
| Var33 | 0.150796704 |

**Model Coefficients for CMS Hierarchical Model (TKA/THA)**

| (Intercept) | -4.0316433 |
| --- | --- |
| Var1 | 0.035679265 |
| Var2 | -0.148849333 |
| Var3 | 0.08807725 |
| Var4 | 0.25290998 |
| Var5 | -0.257380043 |
| Var6 | 0.241117802 |
| Var7 | 0.132427427 |
| Var8 | 0.115796146 |
| Var9 | -0.014769398 |
| Var10 | 0.15814836 |
| Var11 | 0.187159646 |
| Var12 | 0.035368612 |
| Var13 | 0.120886777 |
| Var14 | 0.211303719 |
| Var15 | 0.170049309 |
| Var16 | 0.245655958 |
| Var17 | 0.387470462 |
| Var18 | 0.234387939 |
| Var19 | 0.102192659 |
| Var20 | 0.187162267 |
| Var21 | 0.20830413 |
| Var22 | 0.084196245 |
| Var23 | 0.206829049 |
| Var24 | 0.096990789 |
| Var25 | 0.103278456 |
| Var26 | 0.256166878 |
| Var27 | 0.069134573 |
| Var28 | 0.601609967 |
| Var29 | 0.294460582 |
| Var30 | 0.109616297 |
| Var31 | 0.003492241 |
| Var32 | 0.030762491 |
| Var33 | 0.117221489 |
| PredCluster2 | 0.132189893 |
| PredCluster3 | 0.074260901 |
| PredCluster4 | 0.096335076 |
| PredCluster5 | 0.225685704 |
| PredCluster6 | 0.166772439 |
| PredCluster7 | -0.034196246 |
